# Biogeochemical impacts of fish farming on coastal sediments: Insights into the functional role of cable bacteria

**DOI:** 10.3389/fmicb.2022.1034401

**Published:** 2022-12-22

**Authors:** Diana Vasquez-Cardenas, Silvia Hidalgo-Martinez, Lucas Hulst, Thorgerdur Thorleifsdottir, Gudmundur Vidir Helgason, Thorleifur Eiriksson, Jeanine S. Geelhoed, Thorleifur Agustsson, Leon Moodley, Filip J. R. Meysman

**Affiliations:** ^1^Department of Biotechnology, Delft University of Technology, Delft, Netherlands; ^2^Geobiology, Department of Biology, University of Antwerp, Antwerp, Belgium; ^3^RORUM ehf, Reykjavík, Iceland; ^4^NORCE Norwegian Research Centre, Randaberg, Norway

**Keywords:** electrogenic sulfide oxidation (e-SOx), long-distance electron transport (LDET), aquaculture, sulfur cycling, cable bacteria

## Abstract

Fish farming in sea cages is a growing component of the global food industry. A prominent ecosystem impact of this industry is the increase in the downward flux of organic matter, which stimulates anaerobic mineralization and sulfide production in underlying sediments. When free sulfide is released to the overlying water, this can have a toxic effect on local marine ecosystems. The microbially-mediated process of sulfide oxidation has the potential to be an important natural mitigation and prevention strategy that has not been studied in fish farm sediments. We examined the microbial community composition (DNA-based 16S rRNA gene) underneath two active fish farms on the Southwestern coast of Iceland and performed laboratory incubations of resident sediment. Field observations confirmed the strong geochemical impact of fish farming on the sediment (up to 150 m away from cages). Sulfide accumulation was evidenced under the cages congruent with a higher supply of degradable organic matter from the cages. Phylogenetically diverse microbes capable of sulfide detoxification were present in the field sediment as well as in lab incubations, including cable bacteria (*Candidatus* Electrothrix), which display a unique metabolism based on long-distance electron transport. Microsensor profiling revealed that the activity of cable bacteria did not exert a dominant impact on the geochemistry of fish farm sediment at the time of sampling. However, laboratory incubations that mimic the recovery process during fallowing, revealed successful enrichment of cable bacteria within weeks, with concomitant high sulfur-oxidizing activity. Overall our results give insight into the role of microbially-mediated sulfide detoxification in aquaculture impacted sediments.

## 1 Introduction

Fish comprises an important source of animal protein to the human population, with the global fish production estimated at 177.8 million tons in 2020 (FAO, 2022). To meet this demand, fish farming has significantly expanded over the past decades, accounting for nearly half of the global production (87.5 million tons). A sizeable fraction of this fish farming (33.1 million tons) occurs in marine waters (FAO, 2022). This intensive aquaculture practice generates large quantities of organic waste that predominantly sinks to the seafloor, which includes both waste products (feces) and undigested fish food (Quiñones et al., 2019; Miranda et al., 2020). The enrichment of organic matter in sediments underneath fish cages promotes anaerobic mineralization, and particularly stimulates sulfate reduction, which generates high concentrations of sulfide in sediments (Holmer and Kristensen, 1996). Free sulfide may diffuse out of the sediment into the overlying water and upon oxidation, it can contribute to the depletion of oxygen in bottom waters (la Rosa et al., 2001; Bissett et al., 2006; Choi et al., 2018). When this situation persists, oxygen becomes fully depleted, and free sulfide accumulates in the water column, in a process called euxinia. Due to the intrinsic toxicity of free sulfide to eukaryotes, euxinia events may induce mass-mortality in both benthic and pelagic fauna (Pearson and Rosenberg, 1978; Holmer et al., 2007). Sedimentation of fecal pellets further serve as a dispersion mechanism for pathogens and fish gut-microbes such as *Pseudomonas*, *Vibrio*, and *Clostridia* both in the water column and sediment near the cages (Kolda et al., 2020; Quero et al., 2020).

The increased sedimentation of organic-rich particles near the fish cages, but also to some extent further away due to transport by currents, has a lasting impact on the local sediment geochemistry, microbiology, and faunal communities (Carroll et al., 2003; Holmer et al., 2007; Ballester-Moltó et al., 2017). To safeguard the environmental quality of local marine coastal areas, environmental standards are enforced upon fish farming activities. One widely enforced remediation measure is fallowing, where farming areas must remain inactive for at least 3 months after each production cycle. This process enables the seabed to partially return to its state prior to the on-set of farming (McGhie et al., 2000; Carroll et al., 2003). However, the complete restoration of fish farm impacted sediments can require multiple years, with only an initial recovery observed in the first and second year. This restoration trajectory depends not only on the fish farming practices but also on the prevalent environmental conditions (Pereira et al., 2004; Keeley et al., 2017; Verhoeven et al., 2018). Other, but less-used remediation techniques include: sediment removal, harrowing, re-suspension, addition of detritivores or polychaetes, and sediment irrigation with oxygenated surface water (Kunihiro et al., 2011; Keeley et al., 2017; Ape et al., 2019). The best course of action to constrain the environmental effects of fish farming remains a subject of debate.

In natural environments with highly organic-enriched sediments, sulfur-oxidizing microbes can act as “ecosystem engineers” that counteract euxinic conditions through highly efficient sulfide oxidation. Off the coast of Namibia, pelagic *Gammaproteobacteria* and *Campylobacterota* (formerly known as *Epsilonproteobacteria*) can fully oxidize sulfide in euxinic bottom waters with nitrate, thus potentially preventing or reducing mass mortality events of the fish community (Lavik et al., 2009). Similarly, high abundances of sulfur-oxidizing microbes from the *Campylobacterota* (*Sulfurimonas* and *Sulfurovum*) and mat-forming *Gammaproteobacteria* are typically observed in fish farm impacted sediments (Kawahara et al., 2009; Verhoeven et al., 2018; Kolda et al., 2020; Quero et al., 2020). However, it is presently unclear to what extent these natural microbial communities can help mitigate the impact of fish farming on the seabed, and accelerate the recovery of ecosystem functions in the seabed. The microbially-mediated process of sulfide oxidation is of critical importance in this matter. Sustainable management of fish farming activities hence calls for a better understanding of microbial sulfide oxidation in the seabed.

A highly efficient form of sulfide detoxification was recently discovered in coastal sediments worldwide, which is conducted by cable bacteria performing electrogenic sulfide oxidation (e-SOx; Pfeffer et al., 2012; Malkin et al., 2014; Burdorf et al., 2017). In this unique bacterial metabolism, sulfide oxidation is supported by long-distance electron transport over centimeters (Nielsen and Risgaard-Petersen, 2015; Meysman, 2018). Cable bacteria activity stimulates various other microbial processes, such as sulfate reduction, denitrification, and chemoautotrophy (Vasquez-Cardenas et al., 2015; Kessler et al., 2019; Sandfeld et al., 2020; Liau et al., 2022) promotes cryptic sulfur cycling, and alters the cycling of phosphorous, manganese and heavy metals in sediment (Rao et al., 2014; van de Velde et al., 2016). e-SOx can also play a crucial role in the prevention of euxinic bottom waters. This process was first demonstrated in the organic-rich sediments of a seasonally hypoxic basin and occurs through a sequence of biogeochemical steps (Seitaj et al., 2015). Firstly, the development of e-SOx strongly decreases the pH in anoxic sediments, which dissolves the ambient pool of iron sulfides. The ferrous iron that is liberated diffuses upward and generates a buffering layer of sedimentary iron oxides just below the oxic surface, which is referred to as an iron firewall (Seitaj et al., 2015). When the bottom water turns anoxic, this iron firewall captures the sulfide diffusing upward and this can prevent/delay the escape of sulfide from the sediment over a period of weeks (Seitaj et al., 2015). In doing so, the detrimental effects of hypoxia and euxinia, both in the benthic and pelagic environments, are alleviated naturally (Marzocchi et al., 2018; Hermans et al., 2020). The reduction of sulfide levels in sediments by e-SOx likely favors the growth of seagrass and mussel beds, and the re-colonization of benthic macro- and meiofauna (Seitaj et al., 2015; Malkin et al., 2017; Martin et al., 2018; Bonaglia et al., 2020).

Here, we hypothesize that a diverse microbial community of sulfur-oxidizers, including cable bacteria, could potentially contribute to sulfide detoxification in fish farm sediments during fallowing, thus enhancing the overall recovery process. To investigate this hypothesis, we studied the *in situ* geochemistry and microbial communities in sediments near fish cages on the East coast of Iceland with different production regimes (short versus long production periods). Accompanying laboratory incubations gave additional insight into the recovery process of fish farm sediments and the potential role of cable bacteria.

## 2 Materials and methods

### 2.1 Study area and sample collection

In Iceland, Atlantic salmon (*Salmo salar*) is the most important farmed fish species, and the largest part of its production cycle occurs in sea cages. Our study area was located at two farming sites, Glímeyri and Svarthamarsvik, in Berufjörður, which is a 20 km long and 3–5 km wide fjord on the east coast of Iceland (Supplementary Figure 1). Cages have a 160 m circumference, a depth of 32 m and a resulting volume of 47,000 m^3^, and are arranged in clusters (7 in Glímeyri and 6 in Svarthamarsvik). Each production cycle lasts up to 36 months, depending on temperature, growth rate, and the size of smolts (young salmon) initially stocked. Harvesting starts when maximum biomass is reached with an average fish weight close to 6 kg. Sampling was conducted on September 24th and 25th 2018 on both locations. The temperature of the bottom water was 4°C and salinity was 33. At the time of sampling, the Glímeyri cluster was early in its production cycle (22nd week), and as a result, it still contained a relatively low biomass (∼540 tons divided over 7 cages). In contrast, the Svarthamarsvik cluster was much later in the production cycle (122 weeks) and had already reached the harvesting stage. Hence salmon biomass was much larger (∼2,800 tons in 6 cages). The two sampling sites investigated therefore represent two scenarios, the Glímeyri site with low biomass and a short production period (SPP) and the Svarthamarsvik site with high biomass and a long production period (LPP).

In Berufjörður, the tidal current comes into the fjord from the north and exits to the south (Supplementary Figure 1). We established a transect in parallel to the tidal current from the south-eastern most cage within each of the two clusters. Three sampling sites were selected along each transect: next to the cage (0 m), 50 and 150 m away from the fish cage (Supplementary Figure 1 and Supplementary Table 1). Intact sediment cores were retrieved with a single-core gravity corer (Uwitec) using polycarbonate core liners (60 mm inner diameter and 60 cm length). Four replicate cores were collected per site. All sediment cores were inspected upon retrieval, and only cores with a visually undisturbed sediment surface, and with overlying water (>5 cm) were retained. Cores were first kept in the shade onboard ship (air temperature 5°C), and within 5 h after collection, they were brought to a climate-controlled room at 0°C for further processing.

Overlying water in each core was aerated via an aquarium air pump and cores were kept at 0°C. Microsensor depth profiles were collected for all replicate cores within 24 h, and two cores per site were subsequently sectioned in six layers (slicing at 0.5, 1.0, 1.5, 2.0, 3.0, and 5.0 cm depth). Sediment slices were collected in petri dishes, homogenized, and immediately transferred to Eppendorf tubes which were either immediately frozen at −20°C for DNA analysis (1.5 ml) or fixed with ethanol 96% at a 1:1 ratio (1 ml) for Fluorescent *in situ* Hybridization (FISH).

### 2.2 Laboratory sediment incubations

Laboratory sediment incubations were additionally performed to mimic the recovery process of fish farm sediments during fallowing. To this end, the top flocculent layer and big shells were removed from the sediment cores retrieved from the cage site at both the SPP and LPP sites. This removal of organic-rich top sediment simulates a potential remediation technique considered by the fish farming industry, as well as natural dispersion by waves and currents. The subsequent sediment layer (∼5 cm thickness) was collected in dark plastic jars (250 ml) and transported to the lab, where it was kept in the fridge (4°C) for up to 2 months. Sediments were sieved (350 μm), homogenized, and repacked into plastic core liners with a stopper at the bottom (diameter 2.5 cm; length 4 cm; 4 replicas per site). Cores were kept submerged in aerated artificial seawater (33 salinity, Instant Ocean Sea Salt), incubated at 18°C, and microsensor depth profiles were collected weekly. A few days after the initial appearance of the fingerprint of electrogenic sulfide oxidation (as confirmed by microsensor profiling), one core for each site was sliced in three layers: 0–0.5, 0.5–1.0, and 1.0–3.0 cm. This occurred on day 15 for SPP and on day 59 for LPP. Sediment was homogenized and subsampled as described above for molecular analysis of the microbial community.

### 2.3 Microsensor depth profiling

Geochemical characterization of sediments was done via microsensor profiling (O_2_, H_2_S, and pH) using commercial microelectrodes (Unisense, Denmark). *In situ* cores were measured within 24 h after sediment collection, while laboratory incubations were measured at several time points along the incubation period: 5, 8, 12, 15, 18, 22, 37, 54, and 59 days.

Oxygen depth profiles (tip size, 50 μm) were recorded at 50–100 μm resolution. Depth profiles for H_2_S and pH (tip size, 50 and 200 μm, respectively) were recorded at 200 μm resolution in the oxic zone and at 500 μm depth resolution below. Calibration of O_2_, pH, and H_2_S electrodes was performed as previously described in Malkin et al. (2014). ΣH_2_S was calculated from H_2_S profiles based on pH measurements at the same depth using the R package AquaEnv (Hofmann et al., 2010). Diffusive oxygen uptake (DOU) was calculating using Fick’s first law as follows

$$J\,i=\frac{\varphi }{1-2\,l\,n\,\varphi }*D\,i\,\left(S,T\right)*\frac{\partial C\,i}{\partial x}$$

where φ is the measured porosity (0.8), the term (1–2lnφ) accounts for the tortuosity of the sediment (Boudreau, 1996), D_i_ is the diffusion coefficient of oxygen at the measured temperature (T_field_ = 0°C, T_lab_ = 18°C) and salinity (S = 33 salinity) using the R package marelac (Soetaert et al., 2014). The maximum concentration gradient ($\frac{\partial C\,i}{\partial x}$) was obtained as the linear slope of the concentration profile immediately below the sediment-water interface. Statistical difference between DOU from the different sites was evaluated with ANOVA considering factors such as length of the production period and distance from cage.

### 2.4 Organic matter

Three proxies were used to trace the impact of aquaculture on the organic matter cycling in the sediment at the field sites: (1) stable isotopic signatures of organic matter (δ^13^C_TOC_), under the assumption that feeding pellets, and therefore aquaculture derived organic carbon, have a stable isotope signature distinct from autochthonous marine organic material (McGhie et al., 2000; Holmer et al., 2007); (2) Total organic carbon content (TOC) may be enhanced due to input from the cages depending on prevailing hydrography (Maldonado et al., 2005; Giles, 2008); (3) Biological oxygen demand (BOD) is enhanced by the input of bioavailable aquaculture derived organic matter (Pereira et al., 2004; Piedecausa et al., 2012). To study these three proxies, sediment cores were collected at the cage from each cage cluster, sliced onboard the ship in five distinct layers, and then transported in a cooling box (5°C) to the laboratory for further analysis. For the SPP cage, the surface layer (0–2 cm) and 4 subsurface centimeter-thick layers (5–6, 10–11, 15–16, and 20–21 cm deep) were collected. At the LPP site, the presence of shells only allowed for two-centimeter-thick layers (0–2, 4–6, 8–10, 14–16, and 18–20 cm deep). Measurements of TOC and δ^13^C_TOC_ were done in triplicate for the top layer and once for the subsurface sediments. In the lab, sediment was lyophilized, and a subsample was acidified to determine TOC and δ^13^C_TOC_ simultaneously on an Element Analyzer-Isotope Ratio Mass Spectrometer (EA-IRMS), as described in Kürten et al. (2013).

The BOD was measured in sediment-seawater slurry incubations in small bottle respirometers, i.e., a 100 ml glass bottle with an optic sensor patch glued on the inside of the bottle (Fibox 4 Oximeter, Presens, Germany). This allows for non-invasive measurement of oxygen concentration in the sediment-seawater slurry. Accurate volumes of sediment were sampled by filling a cut off syringe and transferring surface sediment (0–2 cm) to the BOD respirometer. The weight of the bottle before and after sediment addition was noted for accurate calculation of solid sediment volume and sediment water content. Subsequently, the bottle was half filled with filtered seawater (fjord water from NORCE mesocosm facilities) and aerated (using an aquarium air pump and long sterile needle) for 30 min. This was done to reduce potential artifacts of BOD, including oxidation of reduced compounds in the porewater. After air flushing, bottles were filled with filtered seawater to the brim using a plastic flotation disk to prevent loss of sediment and weighed again (to determine the added water volume). Bottles were then sealed with a cap fitted with a Viton septum (ensuring the absence of air bubbles), vigorously shaken and the time-zero (start) oxygen concentration was measured. Sediment slurries were mixed constantly on a rotator and oxygen concentrations were measured at regular intervals during the incubation period that ranged from 5 to 300 min to obtain a sufficient decrease in oxygen content. The oxygen consumption rate was determined through linear regression. BOD (expressed in μmol O_2_ ml sediment^–1^ hr^–1^) was calculated by multiplying the regression slope (μmol O_2_ ml water^–1^ hr^–1^) by the volume of water added (ml) and dividing by the volume of sediment (ml of sediment). All incubations were conducted at 5°C in the dark.

### 2.5 Sediment grain size and porosity

Sediment from each site was collected with a Van Veen grab, collected in buckets and transported to the lab. An aliquot of sediment (∼300 g) was submersed in water for a few hours and then sieved through seven different sieves from 4.0 to 0.063 mm, and run off was collected. All eight sediment fractions were then dried to constant weight at 50°C and grain size was determined as described in Folk and Ward (1957). From lab incubations, sediment was collected for porosity determination from water content and dry solid phase density measurements after sediment was dried to constant weight at 70°C.

### 2.6 DNA extraction and sequencing

Total genomic DNA extraction was performed following the protocol of Zhou et al. (1996) as amended and described in Geelhoed et al. (2020). For field sediments, three depths were selected for DNA extraction: 0–0.5 cm (layer 1), 0.5–1 cm (layer 2), and 3.0–5.0 cm (layer 6). These three layers encompass the organic-rich top layer (top 1 cm), and a deeper anoxic layer, with less organic matter acting as a reference. For the laboratory incubated sediments, only the upper two sediment layers were analyzed as they span the layer in which cable bacteria activity was evident (see below). DNA purity was evaluated via the adsorption ratios at 260/280 nm and 260/230 nm (Ultraspec 2100 spectrophotometer), DNA concentration was assessed with the Qiagen powersoil pro kit on a Qubit 4 fluorometer, and DNA quality was determined on an Implen NP80. DNA concentrations were at least 10 ng/μl. Samples were sent to Novogene Ltd. (Hong Kong, China) for amplicon sequencing of the V3–V4 region of the 16S rRNA gene (position 341–806 bp) using the Illumina paired-end platform with 250 base pair paired-end reads. Primer sequences used were 5′-CCTAYGGGRBGCASCAG-3′ and 5′-GGACTACNNGGGTATCTAAT-3′ (Takahashi et al., 2014).

Raw forward and reverse reads were analyzed using the dada2 R-package (version 1.17.0) that results in a list of Amplicon Sequence Variants (ASVs) with the corresponding counts and taxonomic classification (Callahan et al., 2016). The dada2 pipeline first evaluates the quality of the reads, then filters and trims the reads [trimming parameters: truncLen = (226,223), maxN = 0, truncQ = 2 maxEE = (2,2)]. Forward and reverse reads are merged and chimeras removed using the consensus method. Taxonomy was assigned to ASVs up to genus-level according to the Silva small subunit rRNA reference database v1.38.1 (Quast et al., 2013). Final filtering of the ASV data matrix (dada2 output) was performed by removing singletons, doubletons, and short reads (<400 bp) prior to statistical analysis. Sequences for each sediment layer are available in the NCBI database under BioProject PRNJNA911159, accession numbers SAMN32163971 to SAMN32163991.

### 2.7 Statistical analysis of amplicon sequence variants

The filtered ASV data matrix was used to analyze the microbial community composition. ASV abundance was treated as compositional data and normalized with the variance stabilization transformation (vst) as part of the DESeq2 R-package (version 1.28.1, Anders and Huber, 2010). To assess the overall effect of the fish farms on the *in situ* microbial community a Principal Coordinate Analysis (PCoA) was performed, followed by an Analysis of Similarities (ANOSIM) of the identified sample clusters with a Bray–Curtis similarity matrix and 999 permutations (vegan R-package version 2.5.7; Clarke, 1993). Quantitative analysis of differential expression (DESeq) of ASVs abundances was then used to identify significantly distinct microbes for the sample clusters obtained in the PCoA (Anders and Huber, 2010). DESeq uses a two-way comparison assuming a negative binomial distribution model that returns the log-fold change (LFC), i.e., (logarithmic) difference in the abundance of each ASV between two factors (Love et al., 2014). ASVs were considered differentially abundant when the LFC was ≥|4| and *p* < 0.01. In addition, a non-parametric Spearman correlation analysis was implemented to compare the microbial communities between *in situ* cage and laboratory sediment (microbiomeSeq R package). Only ASVs that were present in at least 30% of the samples were used for the correlation analysis. Correlation index was calculated at the ASV level and reported as significant for *p* < 0.001.

### 2.8 Cable bacteria identification and diversity

The presence of cable bacteria in the fish farm sediments was examined via three approaches. First, scanning electron microscopy (SEM) was used to visualize the unique external morphology of cable bacteria (parallel ridges that run continuously along the filament; Pfeffer et al., 2012). To this end, microscopic sediment chambers were prepared as described in Bjerg et al. (2018). After 2 days, long filaments were observed extending from the sediment toward the edge of the microscopic chamber. Filaments were carefully hand-picked from the chambers with glass hooks, rinsed in miliQ to remove salts, transferred onto polycarbonate filters, air-dried and then sputter-coated with gold. SEM images of the filaments were made with a JEOL 5600 Scanning Electron Microscope (SEM) under low vacuum. Average cell length and width were determined with the Fiji-ImageJ software. This technique was applied to the SPP incubation sediment from day 8.

Secondly, the identity of the filamentous bacteria was investigated with Fluorescence *in situ* Hybridization (FISH). The FISH probe DSB706 was used as it targets most members of the *Desulfobulbaceae* family, including the cable bacteria (Lücker et al., 2007). This probe has previously been used to verify the presence of cable bacteria in a diverse range of sediments (Malkin et al., 2014; Schauer et al., 2014; Burdorf et al., 2017). This FISH method was applied to both field and incubated sediments.

Thirdly, the 16S rRNA gene diversity of cable bacteria sequences was assessed in both field and lab samples. All ASVs assigned to either cable bacteria genera (*Candidatus* Electrothrix or *Candidatus* Electronema*;* Trojan et al., 2016) were pre-filtered by removing ASVs that were not present in at least five samples (>20% of samples) with ≥10 total read counts. Remaining ASV sequences were compiled in MEGA-X (Molecular Evolutionary Genetics Analysis software, version 10.2.2) and the sequence similarity to known cable bacteria 16S rRNA gene sequences was confirmed by querying the GenBank repository via BLASTn. Only ASVs sequences with a nucleotide identity ≥94.5% with either cable bacteria genera were retained. To construct a phylogenetic tree for cable bacteria, 16S rRNA gene sequences (>1,000 bp) were retrieved from GenBank including one sequence for each of the 7 known cable bacteria species and 5 *Desulfobulbus* species as reference sequences (Supplementary Table 2). Additionally, 2 sequences from a potentially new cable bacteria genera [AR-3 and AR-4 (Dam et al., 2021)] and 2 sequences from uncultured bacterium from coastal sediments, that were closely-related to this potential third cable bacteria genus, were also included. In total 36 sequences were aligned with the Muscle software in MEGA-X (Edgar, 2004) and the alignment was manually inspected. The best-fit phylogenetic tree was estimated by IQ-TREE 2 software using maximum likelihood with 1,000 bootstraps via the ultrafast bootstrap approach (Minh et al., 2020). IQ-Tree output resulted in a General time reversible model (GTR) for base substitution rates, with four Gamma rate categories. The consensus tree was visualized with the FigTree (version 1.4.4) and *Escherichia coli* strain U 5/41 16S rRNA gene was used to root the tree.

## 3 Results

### 3.1 Geochemical characterization of fish farm sediments

**FIGURE 1:**
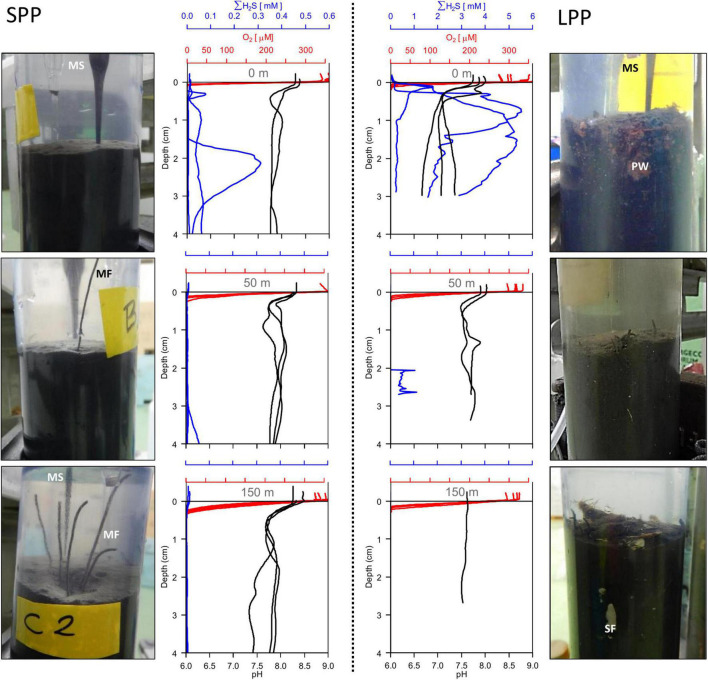
Sediment pictures and microsensor sediment profiles retrieved from two fish farming sites (short and long production period) at 0 m **(top row)**, 50 m **(middle row)**, and 150 m **(bottom row)** away from the cage along the transect. SPP, short production period (Left); LPP, long production period (Right). All replicate profiles are plotted for each station; red, oxygen; blue, total sulfide; black, pH. Photos of sediment cores (diameter: 6 cm) indicate microsensor entering the sediment (MS), particle waste (PW), macrofauna (MF), and shell fragments (SF).

Two salmon farming sites were investigated: Glímeyri had been in operation for 22 weeks (short production period – SPP), while Svarthamarsvik was already 121 weeks in operation (long production period – LPP). Sediments at all sites were classified as very fine-grain sand and silt (with a median grain diameter <63 μm, Supplementary Table 3). Larger particles (>1 cm grain diameter) consisted mainly of shell fragments, and were present at the LPP sites (1.9–6% total weight) and to a lesser degree in SPP sediments (0.3–1.8%). At the SPP sites, the sediment showed a smooth surface, without debris or shells, and active macrofauna was present and increased in number with increasing distance from the cage (Figure 1). In contrast, at the LPP sites, the sediment was black and displayed an uneven surface, with particles of waste feed and large shell debris, and no active macrofauna was apparent in the sediment (Figure 1). Surface TOC values were statistically different along the transect (ANOVA, *p* = 0.004), also when considering both the distance from the cage and the production period (ANOVA, *p* = 0.02). The surface sediment (0–2 cm) at the LPP cage site had the highest TOC (3.33 ± 0.15%) of all sediments, and this surface value was threefold higher than the subsurface TOC at all LPP stations (range: 0.6–1.0%, Table 1 and Supplementary Table 1). At the SPP cage cluster, TOC values were more similar between sites and sediment depths, and subsurface sediments had a higher TOC than at LPP stations (range: 1.2–1.5%, Supplementary Table 1). Stable isotope signatures (δ^13^C_org_) of surface sediment (0–2 cm) were generally more depleted below the cages (−23.4 ± 0.2‰ at SPP and −25.9 ± 0.6‰ at LPP), thus more closely resembling the signature of the fish feed (−25.5 ± 0.4‰, Table 1). Away from the cages, the δ^13^C_org_ signature was closer to that of autochthonous marine organic matter (∼−22.5‰). Significant differences in δ^13^C_org_ were found when considering the distance from cage (ANOVA, *p* = 0.001) and the combined effect of distance and production period (ANOVA, *p* = 0.02). Sediment BOD only significantly varied with distance from cage (ANOVA, *p* = 0.0007). Highest values were recorded for the cage sediments (16–18 μmol O_2_ ml wet sed^–1^ hr^–1^), which decreased by one order of magnitude at 50 m distance (1.0 μmol O_2_ ml wet sed^–1^ hr^–1^) and were lowest at 150 m distance (0.3 and 0.4 μmol O_2_ ml wet sed^–1^ hr^–1^ for the SPP and LPP transects, respectively, Table 1).

**TABLE 1:** Geochemical characteristics of sediments from the field and lab incubations from two fish farm cage clusters: short (SPP) and long (LPP) production period.

	Distance from cage (m)	Conditions	TOC % DW	δ 13C_TOC_ (‰)	BOD μmol O_2_ ml wet sed^–1^ hr^–1^	DOU mmol O_2_ m^–2^ d^–1^	OPD (mm)
SPP	0	Lab incubation (day 8)	NA	NA	NA	23.0 ± 4.8	1.08 ± 0.25
	0	Field	1.7 ± 0.05	−23.4 ± 0.2	18.2 ± 4.6	35.3 ± 6.5	0.74 ± 0.11
	50	Field	1.8 ± 0.08	−22.7 ± 0.1	1.0 ± 0.3	19.2 ± 2.3	1.56 ± 0.46
	150	Field	1.5 ± 0.07	−22.7 ± 0.1	0.3 ± 0.02	11.6 ± 2.4	2.70 ± 0.38
LPP	0	Lab incubation (day 37)	NA	NA	NA	37.5	0.80
	0	Field	3.3 ± 0.1	−25.9 ± 0.6	16.0 ± 4.2	44.2 ± 24.7	0.74 ± 0.31
	50	Field	1.1 ± 0.04	−22.8 ± 0.3	1.3 ± 0.3	16.7 ± 5.4	1.38 ± 0.44
	150	Field	1.1 ± 0.06	−22.5 ± 0.03	0.4 ± 0.4	10.5 ± 2.0	1.76 ± 0.33

TOC, total organic carbon content in dry weight; BOD, benthic oxygen demand; DOU, diffusive oxygen uptake; OPD, oxygen penetration depth.

Given the shell debris in the sediments at LPP, triplicate H_2_S and pH profiling were not possible at 50 and 150 m without damaging the microsensors. The O_2_, H_2_S, and pH depth profiles showed substantial variability within a given site, likely due to the presence of particle waste, macrofauna, and shell fragments that alter the diffusion of solutes in the sediment. Nevertheless, clear differences in porewater chemistry were observed between the two farming sites and with increasing distance from the cages (Figure 1). Sulfide concentrations in sediments underneath the cage were one order of magnitude higher at LPP (5.5 mM) compared to SPP (0.3 mM), and free sulfide was present in the top centimeter of the sediment. However, free sulfide concentrations strongly decreased at 50 m distance for both sites and were below detection limit for the SPP site at 150 m distance. The elevated sulfide concentrations at the LPP cage site were accompanied by more acidic conditions (minimum pH of 6.6 at 3 cm depth). For all other sites pH values remained above 7.2 in the top 5 cm of sediment.

Average diffusive oxygen uptake (DOU) ranged from 10.5 to 44.2 mmol O_2_ m^–2^ d^–1^ and mean oxygen penetration depth (OPD) varied from 0.74 to 2.7 mm across all sediments (Table 1). ANOVA indicated significant differences in DOU with distance from the cages (*p* < 0.0001) but not with the length of the production period (*p* = 0.5). Highest DOU rates were found next to the cages (21.7–91.0 mmol O_2_ m^–2^ d^–1^) and lowest rates at the 150 m sites (8.0–14.2 mmol O_2_ m^–2^ d^–1^). The OPD statistically differed with distance from the cages (ANOVA, *p* < 0.0001) and in combination with the production period (ANOVA, *p* = 0.005). The shallowest OPD was observed at the cage (0.45–1.20 mm) and deepest OPD was found 150 m away from the cage cluster (1.40–3.25 mm). Overall, the impact of the aquaculture activity resulting from enhanced input of degradable organic matter, was apparent in all geochemical variables, with high porewater H_2_S, low subsurface pH, shallow OPD, and high DOU rates near the cages. Although suboxic zones were noted, a clear e-SOx fingerprint (see section “3.3 Geochemical characterization of lab incubations”) was not discernible in any of the depth profiles recorded (*n* = 15; Figure 1).

### 3.2 Microbial community composition in fish farm sediments

In the 21 fish farm sediment samples analyzed (both field and lab incubations) the number of merged reads per sample (without chimeras, nor singletons) ranged from 92,724 to 115,286, which after filtering (removal of doubletons and minimum read length of 400 bp) ultimately resulted in 6,957–10,174 unique ASVs, and 91,542–1,14,619 total number of reads (Supplementary Table 4 and Supplementary Figure 2). Twenty-three phyla had a relative abundance >0.1% with an average of 90% of the total abundance per sample distributed in only six phyla: *Proteobacteria* (13–55%), *Campylobacterota* (8–43%), *Desulfobacterota* (6–23%), *Bacteroidota* (5–11%), *Actinobacteriota* (2–13%), and *Firmicutes* (1–17%). Within the *Proteobacteria* phylum the most prominent Class was *Gammaproteobacteria* (11–38%) followed by *Alphaproteobacteria* (7–33%). The former was especially dominant in LPP lab incubations (0–0.5 cm: 33%, 0.5–1 cm: 11%) because of the high abundance of *Magnetovibrio*. *Pseudomonas* was the most abundant taxon (1–10%) within the *Gammaproteobacteria* class for all samples except for the SPP lab incubation (0–0.5 cm: 17%) where *Thioalkalispira-Sulfurivermis* showed a higher abundance (Supplementary Figure 3). In the *Campylobacterota* phylum, the genus *Sulfurovum* was the most abundant for all sediment samples (5–42%) except for the SPP lab incubation (0–0.5 cm) where *Sulfurimonas* was more abundant (15%, Supplementary Figure 3). Within the *Desulfobacterota* phylum, four classes stand out for their higher abundance: *Syntrophobacteria, Desulfobacteria, Desulfuromonadia*, and *Desulfobulbia* (to which the cable bacteria belong). Of the ASVs classified to genus level, the most abundant were *Desulforhopalus* (1–6%, most abundant in LPP cage sediment), *SEEP*-*SRB4* (1–3%) and *SEEP*-*SRB1* (1–3%) (Supplementary Figure 3). Interestingly, *Ca*. Electrothrix (1.2%) and *Desulfocapsa* (2%) were among the most abundant taxa, in particular for LPP lab incubation (Supplementary Figure 3). The most abundant family within the *Bacteroidota* phylum, across all sediment samples, was *Flavobacteriaceae* (1–5%). Within this phylum three families increased in relative abundance in lab sediments compared to field sediment: *Saprospiraceae*, *NS11-12_marine group*, and *Chitinophagaceae* (particular in LPP lab incubations, data not shown). For the *Actinobacteriota*, two orders were dominant, *Actinomarinales* (1–7%) and *Microtrichales* (1–3%). The family *Lachnospiraceae*, of the *Firmicutes* phylum, was especially present in the *in situ* cage sediments (1–8%, 5 out of 6 samples), and *Streptococcaceae* was more abundant in lab incubations (up to 7% for LPP incubation, 0–0.5 cm, data not shown).

**FIGURE 2:**
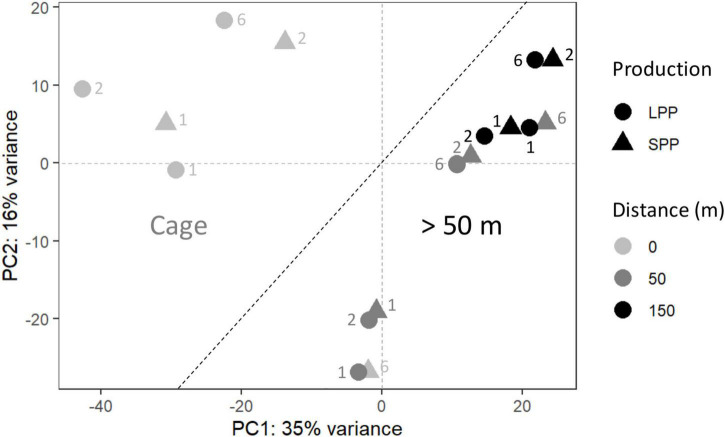
Principal coordinate analysis (PCoA) of the microbial community from fish farm sediment (field samples). The compositional nature of the data was considered by applying a variance stabilizing transformation. Two groups were identified, one for microbial communities from sediment at the Cages (Left), and those found >50 m away from cages (Right). SPP, short production period; LPP, long production period. Sediment layer depth intervals are indicated as follows: 1 = 0–0.5 cm, 2 = 0.5− 1.0 cm, 6 = 3.0–5.0 cm.

To verify the impact of the fish farms on the seabed, differences in the microbial community composition between field sediments were analyzed with PCoA, which explained a total of 51% of the variance in the data. *Distance from the cage* was identified as the main defining factor of the ordination (PC1 = 35%) and differentiated the sediments in two clusters: samples at the fish cages (*Cage*) and samples further away (≥*50 m*) (Figure 2). The deepest sediment layer (3–5 cm depth) from the SPP station at the cage, grouped with the ≥50 m sediment samples. ANOSIM analysis confirmed that the microbial communities differed when considering distance from the cage (Cage vs. >50 m; *R* = 0.6; significance = 0.001), whereas the production period (LPP vs. SPP) had a less significant effect (*R* = 0.4; significance = 0.002). The two PCoA clusters (Cage and ≥50 m) were used in a two-way comparison DESeq analysis to identify the microbial taxa that were differentially abundant between the two clusters. DESeq analysis identified 39 differentially abundant taxa for the Cage cluster and 18 taxa for the >50 m cluster (LFC ≥ |4| and *p* < 0.01, Supplementary Table 5 and Supplementary Figure 4). Of the 39 differentially abundant taxa for the Cage cluster, the taxa with the highest relative abundance belonged to the genera *Streptococcus* (up to 15.7%), *Silanimonas* (up to 8.7%), *Sulfurimonas* (up to 4.0%), *Desulfocapsa* (up to 1.9%), *Thioalbus* (up to 1.8%), and two unknown genera from the *Saprospiraceae* (up to 4.8%) and *Sedimenticolaceae* (up to 3%) families (Supplementary Figure 5). The most abundant taxa for the >50 m cluster within the identified 18 differentially abundant taxa were *Pseudomonas* (up to 3.5%), *Sedimentibacter* (up to 2.6%), and two unknown genera within the *Bacteroidetes vadinHA17* (up to 1.7%) and *Prolixibacteraceae* (up to 2.3%) families (Supplementary Figure 5).

### 3.3 Geochemical characterization of lab incubations

**FIGURE 3:**
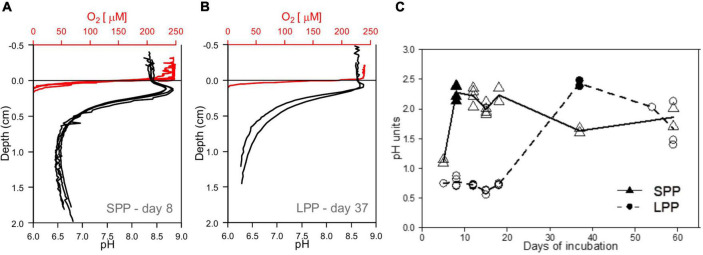
Development of e-SOx in lab incubations with fish farm sediments. **(A,B)** Microsensor profiles showing the presence of e-SOx geochemical fingerprint in incubated cage sediment from both short (SPP) and long production period (LPP). Red, oxygen; black, pH, sulfide not detected. **(C)** Plot of the change in Δ pH in time, with maximum Δ pH obtained at day 8 for sediment from SPP (filled triangle) and day 37 for LPP cage sediments (filled circle). All replicates (points) and average (line) Δ pH are plotted.

Initially, incubated sediments were characterized by a straight pH depth profile (data not shown), which over the course of weeks developed in to the distinct geochemical fingerprint of electrogenic sulfur oxidation (e-SOx) by cable bacteria (Schauer et al., 2014; Meysman et al., 2015). When e-SOx develops, a suboxic zone appears, which separates the oxygen penetration depth (OPD) from the sulfide appearance depth (SAD). At the same time, the pH depth profile shows a maximum in the oxic zone, and then decreases to reach a minimum near the SAD (Nielsen et al., 2010; Meysman et al., 2015). The difference between these two pH values is referred to as ΔpH, and when ΔpH reaches a maximum, it signifies maximum e-SOx activity (Burdorf et al., 2018). Cable bacteria activity was evident in lab incubations from both the SPP and LLP sites yet with a different timing. For the SPP incubations, the maximum ΔpH = 2.3 was reached by day 8 (Figures 3A, C). At this point, the OPD was 1.08 ± 0.25 mm, and DOU was 23.0 ± 4.8 mmol O_2_ m^–2^ d^–1^ (Table 1). In LPP incubations, the e-SOx activity was maximal at 37 days (ΔpH: 2.4, OPD: 0.8 mm, DOU: 37.8 mmol O_2_ m^–2^ d^–1^; Figures 3B, C and Table 1). No free sulfide was detected in the top 2 cm of sediment, throughout the incubation period for both SPP and LPP incubations.

### 3.4 Correlation analysis of microbial communities: Field conditions versus lab incubations

**FIGURE 4:**
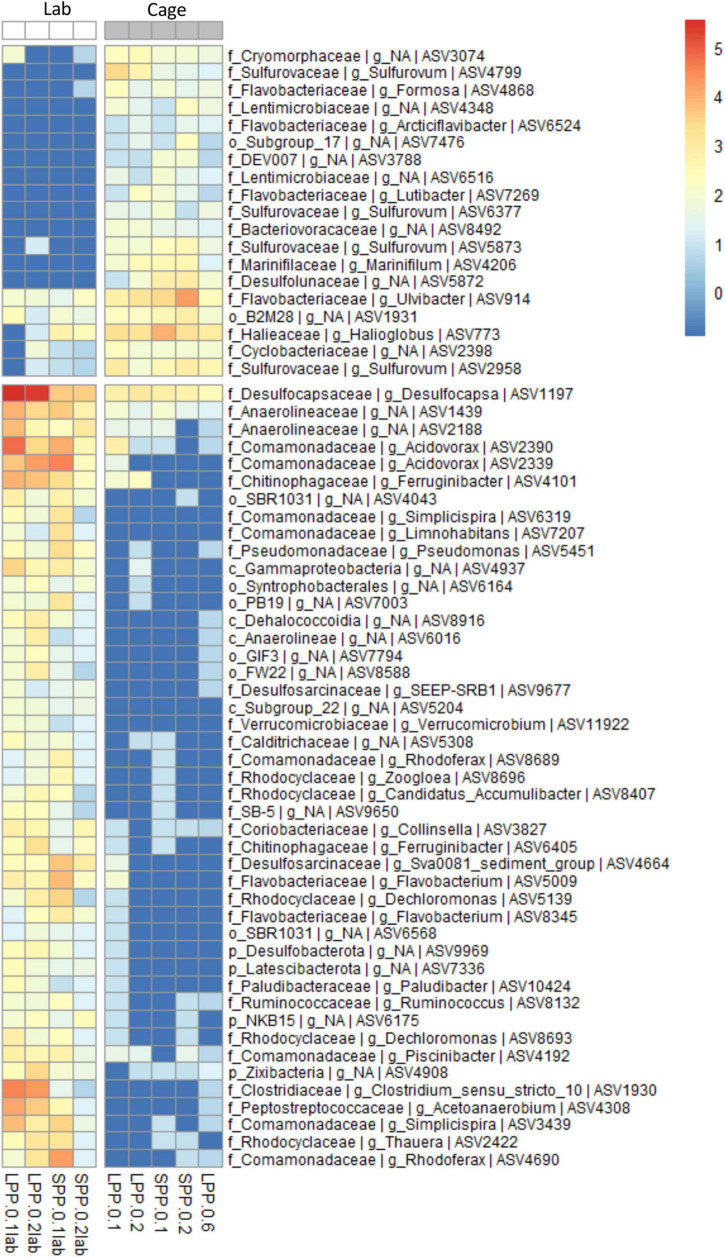
Correlation analysis between the microbial communities in cage sediments from the field and from lab incubations (Spearman correlation, *p* < 0.001). ASV abundance is normalized with variance stabilizing transformation (blue to red scale bar). Genus and next known taxonomic level are stated for each ASV (k, Kingdom; p, Phylum; o, Order; c, Class; f, family; g, Genus). Sample code represent production period: LPP, long; SPP, short; distance from: 0 = at cage; and sediment layer: 1 = 0–0.5 cm, 2 = 0.5–1.0 cm, 6 = 3.0–5.0 cm.

Geochemical profiles of cage sediments from the field and lab incubations differed significantly; in the field high sulfide accumulation was present in porewater that resulted in a surface O_2_-H_2_S interface, whereas a clear e-SOx geochemical signal was obtained in lab incubations (Figures 1, 3A). As such we aimed to identify the differences in microbial community composition between these two sediments. To achieve this the Spearman correlation was used, which measures rank relationships between features. The Spearman coefficient identified a total of 64 ASVs (*p* < 0.001) from the Bacteria kingdom, 19 positively correlated to Cage sediments in the field, and 45 positively correlated to lab incubations with e-SOx (Figure 4). Four ASVs were present in both Cage and Lab sediment: Ulvibacter ASV 914, an unknown genus from the Order B2M28 ASV 1931, *Desulfocapsa* ASV1197 and an unknown genus of *Anaerolineaceae* ASV1439. The first two had the highest abundance of the ASVs significantly correlated to Cage sediments, whereas Desulfocapsa and Anaerolineacea were 10- to 20-fold higher in lab incubations. Additionally, sulfur oxidizing *Sulfurovum* (ASV2958, ASV4799, ASV5873, and ASV6377) correlated to Cage sediments (Figure 4) while *Sulfurimonas* (ASV8, ASV12, and ASV81) and *Thioalbus* (ASV158) correlated to lab incubations (*p* < 0.01, data not shown for *p* > 0.001). All three sulfur oxidizing genera had the highest absolute abundances among the taxa identified with the Spearman’s correlation. The most abundant cable bacteria related ASV (ASV88) correlated to lab incubations with a statistical significance of *p* = 0.02. Within the taxa correlated to lab incubations, *Desulfocapsa* (ASV1197) showed the highest abundance, followed by *Acidovorax* (ASV2390 and ASV2339), while the family *Comamonadaceae* showed the largest number of different taxa (eight ASVs classified to the genera *Acidovorax*, *Limnohabitans*, *Piscinibacter*, *Rhodoferax*, and *Simplicispira*).

### 3.5 Cable bacteria in fish farm sediments

Given the formation of the e-SOx geochemical fingerprint in the laboratory incubations, the associated presence of cable bacteria was verified via three approaches: (1) visualization of handpicked filaments with SEM, (2) taxonomic identification of cable bacteria filaments with FISH, and (3) identification through 16S rRNA gene sequences related to known cable bacteria genera.

**FIGURE 5:**
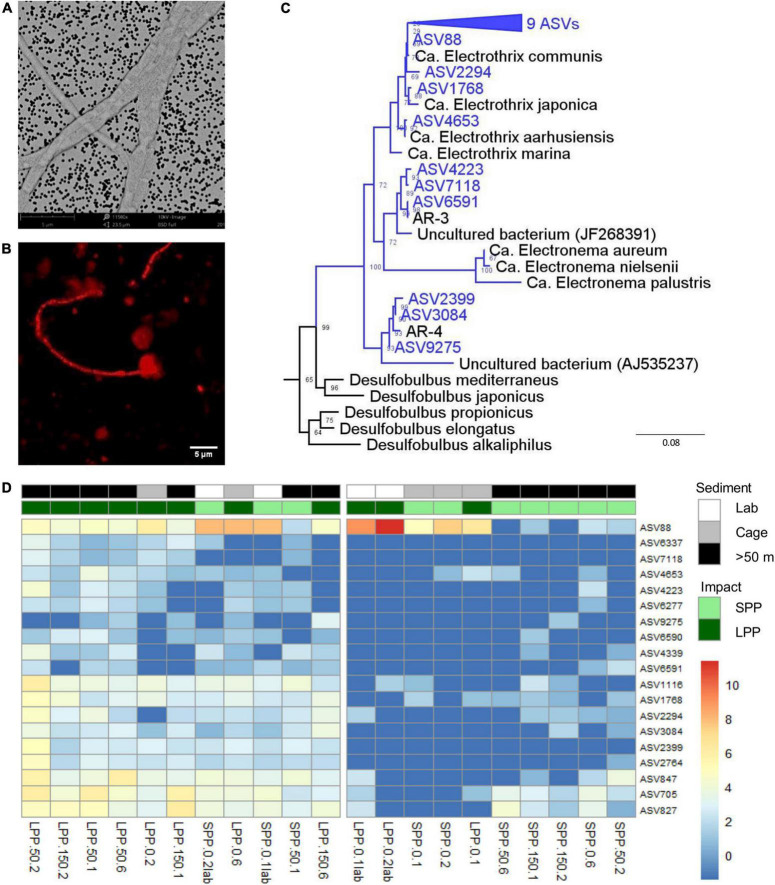
Evidence of the presence of cable bacteria in fish farm sediment. **(A)** SEM image of picked cable bacteria filaments from lab incubated sediments. **(B)** Cable bacteria filament from incubated sediments stained with DSB706 probe via FISH technique. **(C)** Phylogenetic (consensus) tree of 19 ASVs (blue) with >94.5% sequence similarity to known cable bacteria genera (*Ca.* Electrothrix, *Ca.* Electronema, AR-3, and AR-4), from both field and lab incubated fish farm sediments. Bootstrap values are stated at the nodes, 4 references sequences from the Desulfobulbia class are included and *E. coli* was used as root. **(D)** Relative abundance of 19 ASVs identified as *Ca*. *Electrothrix* from all fish farm sediments: Lab incubated sediment (white), field sediment under the Cages (gray), field sediment collected >50 m away from cages (black). Abundances were normalized with variance stabilization transformation (blue to red scale bar). Sample code indicate production period: LPP, long (dark green); SPP, short (light green); distance from cage: 0 = cage, 50 = 50 m, 150 = 150 m; and sediment layer: 1 = 0–0.5 cm, 2 = 0.5–1.0 cm, 6 = 3.0–5.0 cm. Lab incubations are additionally labeled as *lab*.

Unlike any other microbe, cable bacteria have developed a unique conductive wire structure that allows them to “download” electrons from sulfide deep in the sediment, transport them along the filament, and finally “upload” the electrons onto oxygen at the sediment surface (Meysman et al., 2019). These conductive wires lie underneath the outer membrane and are visible as parallel ridges along the filament (Cornelissen et al., 2018). Examination of SEM images from lab incubations (Figure 5A) revealed thin filaments with 10–12 outer membrane ridges (*n* = 10 cells), which is lower than previously observed (range 15–70) but in accordance with thinner filaments (Cornelissen et al., 2018). Filament diameters varied between very thin (0.5 ± 0.1 μm, 13 filaments, 41 cells) and slightly thicker filaments (0.9 ± 0.1 μm, 7 filaments, 21 cells), with an average cell length of 2.8 ± 0.5 μm (*n* = 19 cells).

Secondly, FISH analysis confirmed binding of the specific DNA probe for the *Desulfobulbaceae* family (DSB706) to the observed filamentous bacteria (Figure 5B). Filaments were, however, only identified in subsurface layers of incubated lab sediments (0.5–1.0 cm) and were not detected in field sediments.

The third approach entailed amplicon sequencing, which produces ASVs that can be assigned to the two known cable bacteria genera (Trojan et al., 2016): *Ca.* Electrothrix (marine) and *Ca*. Electronema (freshwater). Combining data from the field and lab incubations, a total of 23 different ASVs were assigned to *Ca*. Electrothrix and none could be assigned to *Ca*. Electronema. Sequences classified as *Ca*. Electrothrix were present in all sediment samples (field and lab), with 19 ASVs present in at least 5 samples with ≥10 total read counts. Thirteen ASVs clustered with *Ca*. Electrothrix species (Figure 5C). The remaining six ASVs clustered outside of the presently known *Ca*. Electrothrix clade but fulfilled the genus-level identity threshold of 94.5% (Yarza et al., 2014) with two other cable bacteria 16S rRNA gene sequences, AR-3 and AR-4, which are described as a potential new cable bacteria genus (Dam et al., 2021, Figure 5C). ASV88, clustering with *Ca*. E. communis, was the most dominant cable bacteria taxon across all samples (Figure 5D). ASV88 accounted for 71–99% of the total cable bacteria abundance for SPP and LPP lab incubations, and >50% for SPP and LPP cage sediment (0–1 cm, Supplementary Figure 6).

Laboratory incubations had the highest relative abundance and sequence counts of cable bacteria related sequences (0.2–1.2%, 229–1,287 sequences) with the highest contributions found in subsurface sediments (0.5–1 cm, Figure 5D and Supplementary Table 4). For field SPP sediments the relative abundance and counts of cable bacteria related sequences were below the minimum values found in laboratory incubations (<0.1%, 20–96 sequences), except for the cage sediment at 0.5–1 cm depth (0.2%, 177 sequences). The relative abundance of cable bacteria sequences in field LPP sediments, was at the lower range found in lab incubations (0.2–0.5%, 185–369 sequences) with the highest contribution of cable bacteria in subsurface sediments (0.5–1 cm depth) at the 50 m away site (Figure 5D and Supplementary Table 4). However, sediment at the LPP cage (0–1 cm) had a lower contribution of cable bacteria sequences (0.1%, 74–116 sequences). The diversity of cable bacteria related ASVs was highest (14–19 ASVs) for SPP lab incubated sediment and all but one LPP sediment (Cage 0–0.5 cm with 4 ASVs, Figure 5D and Supplementary Table 4). Fewer cable bacteria related ASVs (2–10 ASVs) were found in LPP lab incubated sediment and SPP sediments (except for surface sediment from 50 m distance, Figure 5D and Supplementary Table 4).

## 4 Discussion

### 4.1 Overall impact of fish farms on the seabed

In general, aquaculture activities have a strong impact on the sediment inducing geological, biological and/or chemical changes, with the strongest alteration observed below the cages, extending up to several tens of meters away from the fish cages (Carroll et al., 2003; Giles, 2008). As observed in this study, the most conspicuous changes to the sediment occur below the fish cages at the LPP site, where sediment becomes black, with a top layer of non-ingested fish feed, and little to no signs of fauna or bioturbation (Karakassis et al., 2002; Mulsow et al., 2006). Surface sediments were lighter in coloration and less flocculent, with a higher areal abundance of tube worms with increased distance from the cage (Figure 1). An increase in waste particles below the cages, especially at LPP, correlates well with a higher organic carbon content (Table 1; McCaig et al., 1999; Maldonado et al., 2005; Keeley et al., 2012; Ballester-Moltó et al., 2017; Hornick and Buschmann, 2018; Verhoeven et al., 2018). The more depleted stable isotope carbon signature found at cage sediments (Table 1) reflects the accumulation of leftover fish feed that has a depleted stable isotope signature compared to natural marine organic carbon.

The TOC values we obtained in cage sediments (0.7–3.3%) are within the lower range reported for fish farming sediments (2–48%) as reviewed by Giles (2008). Lower OM content can be associated to lengthy fallowing periods and short production periods (Verhoeven et al., 2018) which could explain the values found in the SPP cage sediment (Table 1 and Supplementary Table 1). The degree and extent of the benthic impact, however, can vary as a function of hydrological factors (e.g., current speed and water depth), sediment type (e.g., fine vs. coarse grains), and production characteristics (e.g., production time and fish density) and thus are particular to each environment (Maldonado et al., 2005; Giles, 2008; Grigorakis and Rigos, 2011; Keeley et al., 2012; Verhoeven et al., 2018). The relatively low TOC values found at the LPP cage therefore suggest that other environmental factors, such as strong local currents or mixing due to storms may wash away part of the surface organic-rich, flocculent layer in Berufjörður. In fact, large shell particles were found in the sediment cores collected from the LPP cluster, up to 150 m away, that might have originated from shell fish growth on the cage structure. The increased input of highly degradable organic matter, as indicated by the highest TOC values in top sediment layers at cages (Table 1), may promote the development of chemoorganotrophic microbes such as the differentially abundant *Spirochaete* taxon found in cage sediments (Supplementary Figures 4, 5). *Spirochaete* serve as a bioindicator of highly impacted fish farming sediments (Verhoeven et al., 2018; Quero et al., 2020). Likewise cage sediments were characterized by representatives of the *Cloacimonadales* and *Chitinophagales* orders (up to 1.0 and 4.8% of 16S rRNA sequences, respectively), taxa capable of degrading complex organic compounds and long chain fatty acids such as those present in fish feed and fecal material (Shakeri Yekta et al., 2019).

The observed increase in BOD and DOU at cage sediments relative to sediments further away (Table 1) suggests higher oxygen demand for the mineralization of organic matter (Pereira et al., 2004; Piedecausa et al., 2012). Our oxygen consumption rates are comparable to those found in lab incubations with fish farm sediment (∼30 mmol O_2_ m^–2^ d^–1^), in shellfish farms in the Mediterranean (27–54 mmol O_2_ m^–2^ d^–1^, Dedieu et al., 2007) and in coastal eutrofied areas in Denmark [∼20 mmol O_2_ m^–2^ d^–1^, (Valdemarsen et al., 2014)]. More than 50% of the anaerobic mineralization in active, organic-rich sediments can be accounted for by sulfate reduction which leads to high sulfide production that can accumulate in the porewater of the sediment (Holmer and Kristensen, 1996). Cage sediments at both SPP and LPP sites had the highest concentration of free sulfide in the porewater (Figure 1) confirming the higher mineralization rate at these locations. The appearance of sulfide at the sediment surface in concentrations one order of magnitude higher in LPP vs. SPP cage sediments highlights the effect of fish farms on sulfur cycling.

The microbial community structure follows the geochemical trend described, with a clear difference between cage sediments and those more than 50 m away (Figure 2). In cage sediments, sulfate reduction is likely the predominant mineralization pathway, and 5 ASVs from the class *Desulfobulbia* (*Desulfocapsa, Desulfotalea, Desulfobulbus, MSBL8*, and one unclassified) known to perform sulfate reduction were identified as differentially abundant taxa (Supplementary Figures 4, 5). In parallel, sulfide oxidizing microbes such as *Sulfurovum* (0.6 – 4.0% of 16S rRNA gene sequences), *Magnetospira* (0.04 – 0.5%), and *Thioalbus* (1.2 – 1.8%) also characterized the sulfidic cage sediments (Supplementary Figure 3). All three genera grow (micro)aerobically and oxidize reduced sulfur compounds (sulfide, thiosulfate, tetrathionate and elemental sulfur) in marine environments (Campbell et al., 2006; Takai et al., 2006; Park et al., 2011; Williams et al., 2012; Kojima et al., 2017). In particular, sulfidic fish farm sediments are well-known for an increased presence of *Campylobacterota* (*Sulfurovum* or *Sulfurimonas*) during the production cycle and fallowing periods (Aranda et al., 2015; Verhoeven et al., 2018; Kolda et al., 2020; Miranda et al., 2020; Quero et al., 2020). Thick mats of filamentous microbes from the *Thiotrichaceae* family performing sulfide oxidation are also commonly found in sediments near cages (Aranda et al., 2015; Miranda et al., 2020). However, such microbial mats were not observed at the SPP and LPP field sites upon retrieval of the sediment and taxa assigned to this family contributed less than 0.01% of the 16S rRNA gene sequences. The geochemical fingerprint associated with Beggiatoacea is identified by a decrease pH at the sediment surface and a pH increase at the sulfide appearance depth (Seitaj et al., 2015). This fingerprint was also not observed in sediments from the field nor the lab. Overall, the microbial community in cage sediments had a dominance of sulfate reducing and sulfide oxidizing microbes resembling the community typically found in fish farm sediments (Asami et al., 2005; Kawahara et al., 2009; Choi et al., 2018; Kolda et al., 2020) and in organic-rich marine sediments (Mußmann et al., 2005; Wasmund et al., 2017).

A variety of taxa associated with fish diseases, gut microbiomes and wastewater industry were also found, such as *Streptococcus* in cage sediment, *Pseudomonadales, Bacteroidales, Synergistiales, Cloacimonadales* in sediments >50 m away, and *Pseudomonas, Ruminococcus, Collinsella, Candidatus Accumulibacter*, and *Flavobacterium* in lab incubations (Supplementary Figures 3, 5; Gram et al., 1999; Wexler, 2007; Liu et al., 2015; Møretrø et al., 2016; Ormerod et al., 2016; Martins et al., 2018; Xia et al., 2018). These microbial groups play a key role in anaerobic degradation of organic matter, but due to their pathogenic character, serve as biomarkers for fish farm impacted sediments (Verhoeven et al., 2018; Quero et al., 2020). The presence of potential pathogens and gut-microbiome microbes along the transect studied, highlights the spatial extent of the fish farming activities on the seafloor up to 150 m distance (downstream of prevalent current). The persistence of pathogens surrounding fish farms is one of the main long-term impacts of this industry, that has yet to be resolved (Grigorakis and Rigos, 2011; Martins et al., 2018; Ape et al., 2019).

### 4.2 Studying the recovery of fish farm sediments

In this study, our aim was to provide insight into the geochemistry and microbial communities in sediments impacted by fish farming activities, but also to focus on the recovery effects through dedicated laboratory incubations. One widely implemented method for fish farm sediment recovery is fallowing, in which the input of organic matter is ceased over a period of months to years. Ceasing fish farm activities at a given location results, over time, in a decreased mineralization of organic matter, and hence a decreased production of sulfide through sulfate reduction. The length of this recovery process varies from months to years depending on the fish farming practices and environmental conditions of a specific area (Pereira et al., 2004; Keeley et al., 2017; Verhoeven et al., 2018). To examine whether this recovery process can be accelerated in the lab, two physical disturbances were implemented to fish farm sediments: (i) removal of the organic-rich top sediment and (ii) mixing of sediments. The removal of the organic-rich surface layer prior to incubation can occur naturally as a result of storms and strong currents. Similarly, sediment mixing has been shown to improve sediment quality by reducing the accumulation of sulfide (Keeley et al., 2017), as mixing causes the pool of reduced metals to become partly oxidized (e.g., FeS or FeS_2_ are oxidized to FeOOH). These iron- or manganese (hydr)oxides then become readily available to react with the free sulfide that is being produced via sulfate reduction, thus forming sulfide minerals (e.g., FeOOH combines with H_2_S to form FeS or FeS_2_). Therefore, our laboratory sediment incubations provide insight in to the potential recovery of fish farm sediments during fallowing (ceasing of organic matter input) or those exposed to strong storms (removal of organic-rich sediment and potential mixing of sediment).

The combined effect of no organic matter input, top layer removal and sediment homogenization in the lab incubated sediments resulted in no measurable accumulation of free sulfide in porewater during the entire incubation period (60 days, Figures 3A, B). At the start of the incubation, both SPP and LPP sediments showed a pH depth profile typical for a sediment dominated by anaerobic organic matter mineralization through sulfate reduction (data not shown). Under these conditions there is a constant production of sulfide in subsurface sediments that can lead to the accumulation of sulfide in porewater as observed in *in situ* cage sediments (Figure 1).

Interestingly, after a given period of incubation (8 days in SPP and 37 days in LPP), cage sediments developed the geochemical fingerprint of electrogenic sulfur oxidation (e-SOx) by cable bacteria (Figures 3A, B). This fingerprint is characterized by a pH maximum at the oxygen penetration depth (OPD) and a pH minimum centimeter(s) deeper in the sediment (Nielsen et al., 2010; Pfeffer et al., 2012). Cable bacteria grow naturally and in lab incubations (under similar conditions as the ones performed in this study) in a broad range of sediments where ample sulfide is produced through sulfate reduction or iron sulfide dissolution (Risgaard-Petersen et al., 2012; Malkin et al., 2014; Schauer et al., 2014; Burdorf et al., 2017). The rapid development of cable bacteria in the lab incubations and the presence of *Ca*. Electrothrix related 16S rRNA gene sequences in all sediment samples (both field and in the lab, Figure 5) shows that cable bacteria have the potential to grow in fish farm sediments under conditions that are similar to fallowing. We do note that incubation temperatures in our lab incubations (18°C) were higher than in Icelandic fjords (∼4°C annual average water temperature). Yet, we anticipate that the e-SOx development may still occur within a similar timeframe under field conditions. During incubation of subpolar fjord sediments from Greenland, the e-SOx signature appeared within 26 days of incubation at 0°C (Burdorf et al., 2017), thus demonstrating that low temperatures (∼0°C) do not necessarily delay the development of e-SOx. Under these cold conditions, e-SOx was also sustained for long periods of time (up to 98 days). Thus, the development of e-SOx may still occur within the similar timeframe as in our lab incubations.

Furthermore cable bacteria act as ecosystem engineers by influencing the geochemical cycling in sediments via indirect stimulation of microbial processes such as sulfate reduction, hydrocarbon degradation (toluene, alkanes, and PAHs), iron cycling and denitrification (Müller et al., 2016; Otte et al., 2018; Kessler et al., 2019; Marzocchi et al., 2020; Sandfeld et al., 2020; Liu et al., 2021; Huang et al., 2022; Liau et al., 2022). Indeed, some of the genera that positively correlated to e-SOx activity in our lab incubations (Figure 4) may perform sulfate reduction (*Desulfocapsa, SEEP-SRB1*, and *Sva0081*), denitrification (*Thauera, Dechloromonas, Rhodoferex, Zoogloea*, and *Acidovorax*) and iron oxidation (*Acidovorax*). Cable bacteria further displace the oxic-anoxic interface by gliding up and down through the sediment at a microscale while performing e-SOx (Malkin and Meysman, 2015; Bjerg et al., 2016; Scilipoti et al., 2021). Therefore microbes with the ability to move with and sense the changing interface may also be favored in the presence of cable bacteria. Among the genera strongly correlated to e-SOx (Figure 4) several are known to be aerobic, and have motility and chemotaxis genes (*Dechloromonas, Thauera, Simplicispira*, and *Flavobacterium*). Additionally, *Magnetovibrio*, a motile, microaerophilic, sulfur oxidizer (Bazylinski et al., 2013), increased 20 times in abundance in the lab incubations compared to field sediments (Supplementary Figure 3). Similar positive correlation between *Magnetovibrio* and cable bacteria have been reported in lab incubations, suggesting an adaptive advantage for this microbe potentially linked to the microscale geochemical fluctuations created by cable bacteria activity, in particular related to iron availability (Liau et al., 2022).

The strong O_2_-H_2_S gradients in the sediment, produced by the activity of cable bacteria (Nielsen and Risgaard-Petersen, 2015; Meysman, 2018) have led to a proposed metabolic link, over centimeter depths, between cable bacteria and other sulfur-oxidizing microbes belonging to the *Campylobacterota* or *Gammaproteobacteria* (Vasquez-Cardenas et al., 2015; Lipsewers et al., 2017). *Campylobacterota* inhabit niches with low oxygen and high sulfide, while sulfur-oxidizing *Gammaproteobacteria* tend toward higher oxygen concentrations in environments such as hydrothermal vents and cave waters (Macalady et al., 2008). Steep redox gradients favor the diversification of species, each with their own narrow niche, plausibly related to sulfide:oxygen ratio and the available reduced sulfur compounds (Macalady et al., 2008; Meier et al., 2017; Pjevac et al., 2018). The activity of cable bacteria may therefore have a cascading effect on sulfur cycling that further drives the niche differentiation and co-occurrence of phylogenetically diverse sulfur oxidizing community. In fact, micro-aerophilic, sulfur-oxidizing taxa [*Sulfurimonas* and *Thioalkalispira*-*Sulfurivermis*, (Sorokin et al., 2002; Takai et al., 2006)] were found to increase 9 and 18 times (respectively), exhibiting higher average relative abundances (7.3 and 4.7%, respectively) in our lab incubation compared to field sediments (Supplementary Figure 3). Likewise, a positive correlation between sulfur-oxidizing Gammaproteobacteria (*Thiogranum* and *Sedimenticola*) and cable bacteria was recently observed in sulfidic sediments from seasonally hypoxic area in Chesapeake bay (Liau et al., 2022). The potential for improved recovery of fish farm sediments, via efficient sulfide removal by a diverse microbial community enhanced by cable bacteria, is evident from our study. However, the nature of the interactions between the species and the intensity of the synergetic effects remain to be resolved.

Overall our laboratory incubations suggest that ceasing the input of organic matter (fallowing) and removing organic-rich surface sediment (storms and current) can accelerate the recovery of sediments in fish farm impacted areas. Mixing of anoxic sediments (storms, currents, and fauna) as well as the development of cable bacteria significantly reduce or even prevent the accumulation of free sulfide in the porewater. Moreover, the rapid development of cable bacteria in SPP sediment lab incubations indicates that the implementation of shorter production periods at one location may result in a faster sulfur-detoxification during fallowing.

### 4.3 Absence of strong e-SOx in sediments during the production period

The development of cable bacteria in fish farm sediment incubated in the lab raises the question as to why there was no clear e-SOx geochemical fingerprint in the field during the production cycle, and thus what the functional role of cable bacteria is in fish farm sediments. In coastal marine sediments, the e-SOx signal is typically found in sediments with moderately active sites with sulfide concentrations up to 0.3 mM and DOU rates <35 mmol O_2_ m^–2^ d^–1^ (Malkin et al., 2014; Burdorf et al., 2016, 2017; van de Velde et al., 2016). However, cable bacteria have also been found in sediments with high sulfide concentration (up to 3 mM), high organic matter content (up to 4.4%), and high mineralization rates (35–126 mmol O_2_ m^–2^ d^–1^) such as mussel bed, oyster reefs, mangroves, and seasonally hypoxic basins (Burdorf et al., 2017; Lipsewers et al., 2017; Malkin et al., 2017). In these environments, e-SOx plays a major role in the oxygen consumption and sulfide oxidation of the sediment (Nielsen and Risgaard-Petersen, 2015). The sulfide concentrations next to the LPP cage exceed the higher limit reported for sediments with an e-SOx signal. Thus development of cable bacteria, or that of their associated microbes, may be impeded in these (highly) sulfidic environments. These environmental conditions may rather favor the rapid colonization by microaerophilic, sulfur oxidizing *Sulfurimonas* and *Sulfurovum* that characterize steep redox-gradient environments, with high sulfide concentration (Campbell et al., 2006; Han and Perner, 2015; Meier et al., 2017), as observed in sediment below the cages (Figure 4). The lower sulfide concentrations found in the sediments >50 m away from the cages could therefore potentially allow for the growth of cable bacteria. Indeed, sediments from LPP sites at 50 m distance, and to a lesser degree from SPP, showed a diversity and relative abundance of cable bacteria similar to that found in the lab incubations (Figure 5D and Supplementary Table 4). In sediments with a clear e-SOx signature, cable bacteria can account for 0.6% up to 7% of the total 16S rRNA gene sequences, and as low as 0.2% in oxic layers or in suboxic sediment when the e-SOx signal is weakening (Klier et al., 2018; Otte et al., 2018; Geelhoed et al., 2020; Dam et al., 2021; Liau et al., 2022). A recent study further shows a 10-fold higher percentage of cable bacteria sequences, when comparing DNA-based to RNA-based 16S rRNA genes in the same sediment sample (Liau et al., 2022). Therefore relative abundances below 1%, as the ones obtained here for DNA-based sequences, could still represent high metabolic activity of cable bacteria. The 16S rRNA gene sequence abundance of cable bacteria in our lab incubations (0.2–1.2%) and in LPP sediments >50 m away from the cage (0.2–0.5%; Supplementary Table 4) therefore suggest that e-SOx may be occurring in sediments, although it does not dominate the geochemistry of the sediment. Interestingly, more than one species of cable bacteria was found in all sediment samples, with the highest variety of cable bacteria related ASVs in SPP lab incubations (with e-SOx) and in the LPP field sediments (without e-SOx Figure 5D). The coexistence of several species of cable bacteria at one location has been ascribed to non-exclusive competition between species (Marzocchi et al., 2018). However, apart from salinity, little is known about the factors controlling the diversity patterns of cable bacteria (Trojan et al., 2016; Dam et al., 2021).

The lack of a strong e-SOx geochemical signal in sediments near and surrounding the fish cages may be further attributed to the physical disturbance of the sediment. One of the clearest effects of fish farms is the increase in food particles and fish waste below and around the cages, thus increasing the deposition rate. In addition, the cages themselves serve as artificial surfaces for bivalves and barnacles that need to be regularly removed by divers to ensure good water circulation through the cages. Shell debris is carried by the current downstream where it is deposited on the sediment surface as seen in sediment cores 150 m away from the cages (Figure 1 and Supplementary Table 3). The input of particles (fish feed, fecal pellets, and shells) onto the sediment, disrupts the geochemical gradients and changes the microscale niches available for microbes in the top centimeters of the sediment. Cable bacteria filaments orient and glide vertically in the sediment to maintain a connection between the surface oxygen and the deeper sulfide-horizon (Malkin and Meysman, 2015; Bjerg et al., 2016). However, the rate of particle input in fish farm areas is potentially greater than the speed at which cable bacteria glide or grow, and thus a stable, clear e-SOx signature may be impeded. Moreover, the mixing of sediment by macrofauna bioturbation in the fish farm sediment, as seen in SPP sediments (Figure 1) may also break, disorient, or bury cable bacteria filaments, obscuring the e-SOx geochemical fingerprint (Malkin et al., 2014; Burdorf et al., 2016). The presence of cable bacteria related sequences in all our samples and their development in the laboratory incubation (Figures 3, 5) suggests that cable bacteria may be performing e-SOx to some degree in the field, but because of physical perturbations, this processes does not dominate the geochemistry of the sediment. Alternatively, cable bacteria have the complete sulfate reduction pathway encoded in their genome (Kjeldsen et al., 2019), thus they could use this metabolism to live in subsurface sediments until the appropriate conditions arise for them to grow via e-SOx. Nonetheless, the ability for cable bacteria to perform sulfate reduction has not been physiologically demonstrated. The precise environmental requirements and full physiological characterization of cable bacteria remain one of the main fields for further study of this electrogenic microbe (Burdorf et al., 2017; Kjeldsen et al., 2019; Geerlings et al., 2020, 2021; Scilipoti et al., 2021; Malkin et al., 2022).

## 5 Conclusion

Our study foremost confirms the detrimental geochemical and microbial impacts, both temporal and spatial, of waste particle accumulation on the seabed originating from fish farming activities. A detailed study of the residual organic matter accumulation during repetitive production-fallowing cycles is thus recommended in the SW coast of Iceland to properly constrain the recovery capacity of the benthic system and propose appropriate regulation. Our study suggests that physical manipulation of fish farm sediment such as removal of surface sediment, combined with a period of fallowing (ceased organic matter input) aid in the development of e-SOx by cable bacteria. As a consequence mineralization of organic matter via sulfate reduction may be heightened, as well as the development of potential cooperative interspecies relationships that may further enhance sediment recovery by rapidly consuming sulfide. Therefore we propose that cable bacteria can have a functional role in fish farm sediments, most likely during fallowing periods, resulting in three main remediation processes (1) promotion of efficient sulfur cycling by phylogenetically diverse microbes via interactions with cable bacteria, (2) short-term mitigation of sulfide concentrations via e-SOx due to the rapid consumption of sulfide in the top centimeters of the sediment, thus enabling better colonization conditions for macrofauna, and (3) formation of an iron-firewall which can prevent sulfide build-up in the long-term (Seitaj et al., 2015) once a new production cycle is started at the same location. Our hypothesis awaits *in situ* validation (e.g., occurrence of e-SOx geochemical fingerprint during fallowing) or targeted organic loading experiments in mesocosms. Application of microbial fuel cells has also recently been proposed as an efficient sulfide removal strategy in fish farm sediments (Algar et al., 2020). Interestingly, the use of sediment microbial fuel cells can also induce the appearance of the e-SOx geochemical fingerprint (Reimers et al., 2017; Li et al., 2020; Marzocchi et al., 2020). Potential microbial remediation of sulfidic fish farm sediments may thus be achieved with a combination of physical alterations to sediments, shorter production periods, and microbial fuel cells (if upscaling is feasible, Algar et al., 2020). The synergetic effect of these approaches may lead to more environmentally sustainable practices, with lower long-term adverse impacts, for this growing industry.

## Data availability statement

The molecular data presented in this study are available in the NCBI under BioProject PRJNA 911159.

## Author contributions

DV-C, SH-M, and FM conceptualized the study and in collaboration with LM, GH, TE, and TA were involved in funding acquisition. DV-C, SH-M, TT, GH, TE, and LM went to the field and collected all the samples. LM and DV-C performed the lab incubations and sediment-slurries. DV-C, SH-M, LH, and JSG obtained and processed all microbial and microsensor data. LM and TA performed the grain size, stable isotope, and organic matter analysis. DV-C performed all statistical analysis and wrote the manuscript with contributions from all co-authors. All authors approved the submitted version.
