# Supplementary material for: Biogeochemical impacts of fish farming on coastal sediments: Insights into the functional role of cable bacteria
Source: Front Microbiol. 2022 Dec 22;13:1034401. doi: 10.3389/fmicb.2022.1034401 (PMC9814725; doi:10.3389/fmicb.2022.1034401)
Supplement: Supplementary file 1 [file Data_Sheet_1.PDF]

Supplementary Material

Farming site	Distance from cage (m)	Latitud	Longitud	Water depth	Sediment layer (cm)	TOC	$\delta^{13}C_{org}$
Glimeyri Short production period -SPP	0	64°43,4842	14°23,6048	51.9 m	0-2	1.7	-23.4
					5-6	1.4	-22.9
					10-11	1.3	-22.9
					15-16	1.2	-23.1
					20-21	1.3	-23.1
	50	64°43,4516	14°23,5684	50.0 m	0-2	1.8	-22.7
					5-6	1.5	-23.0
					10-11	1.4	-23.0
					15-16	1.3	-22.8
					20-21	1.3	-22.9
	150	64°43,3930	14°23,5394	46.0 m	0-2	1.5	-22.7
					5-6	1.4	-22.7
					10-11	1.3	-23.0
					15-16	1.2	-22.9
					20-21	1.3	-23.1
Svarthamarsvik Long production period - LPP	0	64°42,2984	14°22,1008	35.0 m	0-2	3.3	-25.9
					4-6	1.0	-23.6
					8-10	0.7	-23.2
					14-16	0.7	-23.0
					18-20	0.6	-23.3
	50	64°42,2587	14°22,0517	29.5 m	0-2	1.2	-22.8
					4-6	0.9	-22.9
					8-10	0.9	-23.0
					14-16	0.7	-22.8
					18-20	0.7	-22.6
	150	64°42,2204	14°21,9782	29.9 m	0-2	1.1	-22.5
					4-6	1.0	-22.8
					8-10	1.0	-23.2
					14-16	0.7	-22.8
					18-20	0.7	-22.6

Supplementary Table 1. Geographical coordinates, distance from cage, and water depth of the studied fish farming sites. TOC and $\delta^{13}C_{org}$ are listed per sediment layers.

Supplementary Material

**Species**	**Strain**	**Genbank accession number**
Candidatus Electrothrix japonica		KR912349
Candidatus Electrothrix communis		KR912347
Candidatus Electrothrix communis		KR912339
Candidatus Electrothrix aarhusiensis MCF4-14		KR912338
Candidatus Electrothrix marina A3		KR912341
Candidatus Electrothrix marina A2		KR912340
Candidatus Electronema aureum		Ga0183576.12823
Candidatus Electronema nielsenii Gib-F1		KP728462
Candidatus Electronema palustris Gib-F3		KP728463
Candidatus Electronema palustris Gib-F4		KP728464
Candidatus Electronema sp cloneS2		MN947610
Candidatus Electronema sp cloneS4		MN947612
Candidatus Electronema nielsenii		KP728465
Desulfobulbus propionicus	DSM 2032	NR042971
Desulfobulbus propionicus	DSM 2032 - 1pr3	NR074930
Desulfobulbus elongatus	FP; DSM 2908	NR029305
Desulfobulbus alkaliphilus	APS1	NR117882
Desulfobulbus mediterraneus	86FS1; DSM 13871	NR025150
Desulfobulbus rhabdoformis	M16	U12253
Desulfobulbus japonicus	Pro1	NR040977
Desulfobulbus mediterraneus	NA62	AJ866934
Escherichia coli	U 5/41; ATCC 11775	NR024570
Uncultured bacterium		AJ535237
Uncultured bacterium		JF268391

Supplementary Table 2. List of sequences and accession numbers included in phylogenetic tree of cable bacteria (Fig 3 main text).

**Farming site**	**Distance from cage (m)**	**Grain size [mm]**							
		**4**	**2**	**1**	**0.5**	**0.25**	**0.125**	**0.063**	**<0.063**
Glimeyri – SPP	0	0.6	0.7	0.5	0.7	4.9	15.8	21.7	55.0
	50	0.2	0.1	0.0	0.1	1.0	6.0	18.6	74.0
	150	0.6	0.0	0.0	0.0	0.5	1.4	19.6	77.7
Svarthamarsvik - LPP	0	1.0	0.6	0.3	0.5	1.2	8.4	24.0	64.1
	50	4.9	0.9	0.7	0.5	0.9	2.9	17.8	71.3
	150	1.4	0.3	0.2	0.4	0.7	3.6	18.6	74.8

Supplementary Table 3. Grain size analysis of the two fish farm cage clusters studied: short (SPP) and long production period (LPP). Percent of total weight is reported for each grain size.

Sample code	Cage cluster	Distance from cage (m)	Sediment type	Sediment			Total number of reads – output dada2 pipeline								Filtered ASV data matrix		ASVs assigned to Ca. Electrothrix		
				Depth (cm)	Layer	Grams	input	filtered	merged	Merged /input	nonchim	Nonchim /merged	Non singletons	Non singletons / merged	Total # reads	No. of ASVs	Number	Counts	Rel. abund
SPP.0.1lab	SPP	0	Lab 15 days	0-0.5	1	0.64	149999	136126	120821	81%	105804	88%	101520	84%	99996	7870	15	253	0.2%
SPP.0.2lab				0.5-1.0	2	0.58	137900	124741	110890	80%	98752	89%	95271	86%	94055	8551	15	401	0.4%
LPP.0.1lab	LPP		Lab 59 days	0-0.5	1	0.65	144590	128984	120578	83%	116764	97%	115286	96%	114619	7311	5	229	0.2%
LPP.0.2lab				0.5-1.0	2	0.63	136715	124065	114254	84%	109493	96%	107753	94%	106721	8002	2	1287	1.2%
SPP.0.1	SPP	0	Field	0-0.5	1	0.49	140280	127957	118464	84%	111226	94%	109711	93%	109268	7179	3	29	0.03%
SPP.0.2				0.5-1.0	2	0.44	130697	119898	111052	85%	104039	94%	102535	92%	102079	7414	2	177	0.2%
SPP.0.6				3.0-5.0	6	0.49	145727	132429	119411	82%	106189	89%	103565	87%	102871	7693	9	39	0.04%
SPP.50.1		50		0-0.5	1	0.63	139188	126615	116651	84%	108897	93%	106937	92%	106202	8380	16	96	0.1%
SPP.50.2				0.5-1.0	2	0.61	139456	127263	118146	85%	111885	95%	110061	93%	109310	9045	10	39	0.04%
SPP.50.6				3.0-5.0	6	0.55	148283	135573	120087	81%	105287	88%	101530	85%	100452	8125	4	37	0.04%
SPP.150.1		150		0-0.5	1	0.52	141038	127213	117325	83%	110647	94%	108876	93%	108178	8319	9	30	0.03%
SPP.150.2				0.5-1.0	2	0.57	143388	129261	115495	81%	101047	87%	97458	84%	96462	7978	7	20	0.02%
LPP.0.1	LPP	0	Field	0-0.5	1	0.53	146118	134720	124745	85%	113918	91%	111848	90%	111275	6957	4	74	0.1%
LPP.0.2				0.5-1.0	2	0.55	131384	116848	108207	82%	101539	94%	99736	92%	99265	7716	14	116	0.1%
LPP.0.6				3.0-5.0	6	0.62	145891	131036	120942	83%	114381	95%	112445	93%	111765	8459	17	369	0.3%
LPP.50.1		50		0-0.5	1	0.77	136016	122459	109288	80%	98627	90%	95117	87%	94120	9122	19	267	0.3%
LPP.50.2				0.5-1.0	2	0.71	135718	122536	112297	83%	105267	94%	103025	92%	102074	9189	18	555	0.5%
LPP.50.6				3.0-5.0	6	0.69	132210	119694	109311	83%	104218	95%	101876	93%	100217	10174	19	244	0.2%
LPP.150.1		150		0-0.5	1	0.53	138302	125289	109604	79%	96921	88%	92724	85%	91542	8827	15	192	0.2%
LPP.150.2				0.5-1.0	2	0.69	144338	129950	118244	82%	111281	94%	108716	92%	107628	9933	17	185	0.2%
LPP.150.6				3.0-5.0	6	0.51	145016	129321	116989	81%	109387	94%	106547	91%	105457	9038	14	137	0.1%

Supplementary table 4. Sample information including cluster, sediment depth, and grams used for DNA extraction. Number of initial reads per sample, output of Dada2 pipeline, and number of reads and unique ASVs remaining after filtering parameters. Number of unique ASVs, counts and relative abundance of cable bacteria related 16S rRNA sequences are listed per sample. Sample code represent Production period: LPP=long, SPP=short; distance from cage: 0= cage, 50= 50m , 150= 150 m; and sediment layers: 1= 0-0.5 cm, 2= 0.5-1.0 cm, 6= 3.0-5.0 cm

Supplementary Material

	Phylum	Class	Order	Family	Genus	ASV	LFC	Padj
> 50 m	Actinobacteriota	Actinobacteria	Euzebyales	Euzebyaceae	NA	ASV3550	4.6	1.4E-03
		Acidimicrobiia	IMCC26256	NA	NA	ASV3971	4.2	6.4E-03
	Bacteroidota	Bacteroidia	Bacteroidales	Bacteroidetes vadinHA17	NA	ASV174	4.8	2.2E-03
					NA	ASV447	6.6	2.7E-03
				Dysgonomonadaceae	Proteiniphilum	ASV1057	5.5	3.0E-03
				Rikenellaceae	DMER64	ASV898	5.6	5.5E-03
				Prolixibacteraceae	NA	ASV133	4.6	6.2E-03
	Caldisericota	Caldisericia	Caldisericales	WCHB1-02	NA	ASV917	6.2	2.2E-03
	Chloroflexi	Anaerolineae	Anaerolineales	Anaerolineaceae	Leptolinea	ASV637	6.6	1.9E-03
	Cloacimonadota	Cloacimonadia	Cloacimonadales	Cloacimonadaceae	W5	ASV1854	4.9	9.6E-03
	Desulfobacterota	Syntrophorhabdia	Syntrophorhabdales	Syntrophorhabdaceae	Syntrophorhabdus	ASV516	4.6	3.3E-03
		Syntrophia	Syntrophales	Smithellaceae	Smithella	ASV796	6.0	7.8E-03
	Firmicutes	Clostridia	Christensenellales	Christensenellaceae	R-7_group	ASV453	4.9	2.3E-03
			Peptostreptococcales	Sedimentibacteraceae	Sedimentibacter	ASV147	4.1	3.9E-03
	Myxococcota	Polyangia	Haliangiales	Haliangiaceae	Haliangium	ASV476	4.6	3.4E-03
	Proteobacteria	Gammaproteobacteria	Pseudomonadales	Pseudomonadaceae	Pseudomonas	ASV55	4.3	1.9E-03
	SAR324_clade(Marine_group_B)		NA	NA	NA	ASV496	4.7	3.3E-03
	Synergistota	Synergistia	Synergistales	Synergistaceae	NA	ASV955	6.0	1.4E-03
Cage	Bacteroidota	Bacteroidia	Chitinophagales	Saprospiraceae	NA	ASV140	-5.1	6.1E-10
	Campilobacterota	Campylobacteria	Campylobacterales	Sulfurimonadaceae	Sulfurimonas	ASV67	-5.4	8.7E-05
	Cloacimonadota	Cloacimonadia	Cloacimonadales	MSBL8	NA	ASV1577	-5.5	1.8E-08
					NA	ASV2703	-5.6	3.9E-06
					NA	ASV2983	-5.3	2.7E-05
					NA	ASV2342	-5.3	3.3E-05
				NA	NA	ASV2085	-5.2	3.2E-06
	Cyanobacteria	Cyanobacteriia	Synechococcales	Cyanobiaceae	Cyanobium_PCC-6307	ASV940	-7.0	4.6E-06
	Desulfobacterota	Desulfobulbia	Desulfobulbales	Desulfocapsaceae	Desulfocapsa	ASV86	-6.0	5.7E-10
						ASV522	-9.7	3.1E-07
				Desulfurivibrionaceae	MSBL7	ASV1332	-5.7	1.9E-05
	Firmicutes	Bacilli	Lactobacillales	Streptococcaceae	Streptococcus	ASV18	-7.3	2.6E-09
					Streptococcus	ASV2531	-5.1	2.2E-03
				Enterococcaceae	Enterococcus	ASV1560	-5.5	7.5E-03
	Myxococcota	Myxococcia	Myxococcales	Myxococcaceae	P3OB-42	ASV3305	-5.2	3.2E-07
	Patescibacteria	Saccharimonadia	Saccharimonadales	NA	NA	ASV8137	-6.4	8.1E-03
	Proteobacteria	Alphaproteobacteria	Acetobacterales	Acetobacteraceae	Roseococcus	ASV1850	-5.1	1.9E-05
			Kordiimonadales	Kordiimonadaceae	Kordiimonas	ASV389	-8.3	1.8E-07
				NA	NA	ASV737	-6.8	8.0E-07
			Rhizobiales	Devosiaceae	NA	ASV761	-7.3	5.7E-06
			Rhodobacterales	Rhodobacteraceae	Amaricoccus	ASV3451	-5.1	1.9E-03
					Cribrihabitans	ASV1582	-5.9	3.4E-04
					Rhodobacter	ASV3645	-5.2	5.3E-04
					Tabrizicola	ASV3046	-8.6	1.1E-03
			Rhodospirillales	Magnetospiraceae	Magnetospira	ASV809	-7.3	5.5E-06
					NA	ASV2650	-5.5	1.9E-03
		Gammaproteobacteria	Burkholderiales	Comamonadaceae	Rivibacter	ASV1778	-5.3	1.0E-06
			Chromatiales	Sedimenticolaceae	NA	ASV142	-6.4	5.9E-09
			Ectothiorhodospirales	Ectothiorhodospiraceae	Thioalbus	ASV117	-7.2	5.5E-12
						ASV499	-5.5	9.7E-09
				NA	NA	ASV1653	-8.3	3.3E-04
			Oceanospirillales	Pseudohongiellaceae	Pseudohongiella	ASV2080	-7.8	4.2E-03
			Steroidobacterales	Woeseiaceae	Woeseia	ASV552	-7.3	2.7E-07
			Xanthomonadales	Xanthomonadaceae	Silanimonas	ASV130	-7.4	5.9E-14
						ASV136	-7.3	1.1E-12
						ASV1830	-6.2	1.6E-05
	SAR324_clade(Marine_	NA	NA	NA	NA	ASV632	-7.0	4.9E-07
	Spirochaetota	Spirochaetia	Spirochaetales	Spirochaetaceae	Spirochaeta_2	ASV5494	-8.1	9.0E-04
	Verrucomicrobiota	Kiritimatiellae	Kiritimatiellales	Kiritimatiellaceae	R76-B128	ASV2754	-5.4	8.3E-06

Supplementary table 5. Taxonomic list of 57 differentially abundant ASVs determined with DESeq analysis for the two PCoA sample clusters: Cage and >50m. LFC: log fold change, Padj: adjusted P-value

Supplementary Material

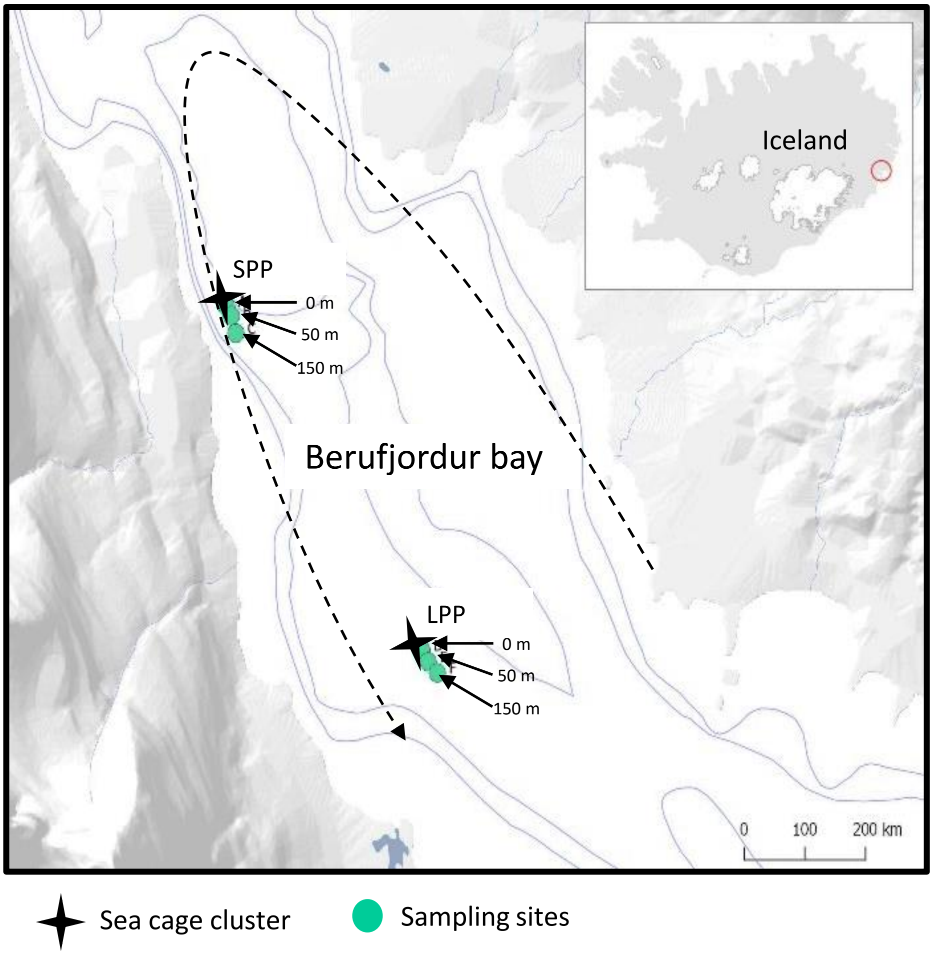

Supplementary figure 1. Location of two sea cage clusters (cross) in Berufjordur Bay on the SW coast of Iceland (red circle in inset map). Sites along the transect (0, 50, 150 m) down-current from the cage clusters are represented with green points, and the direction of the current is represented by the curved arrow. Short (SPP) and long (LPP) production period.

Supplementary Material

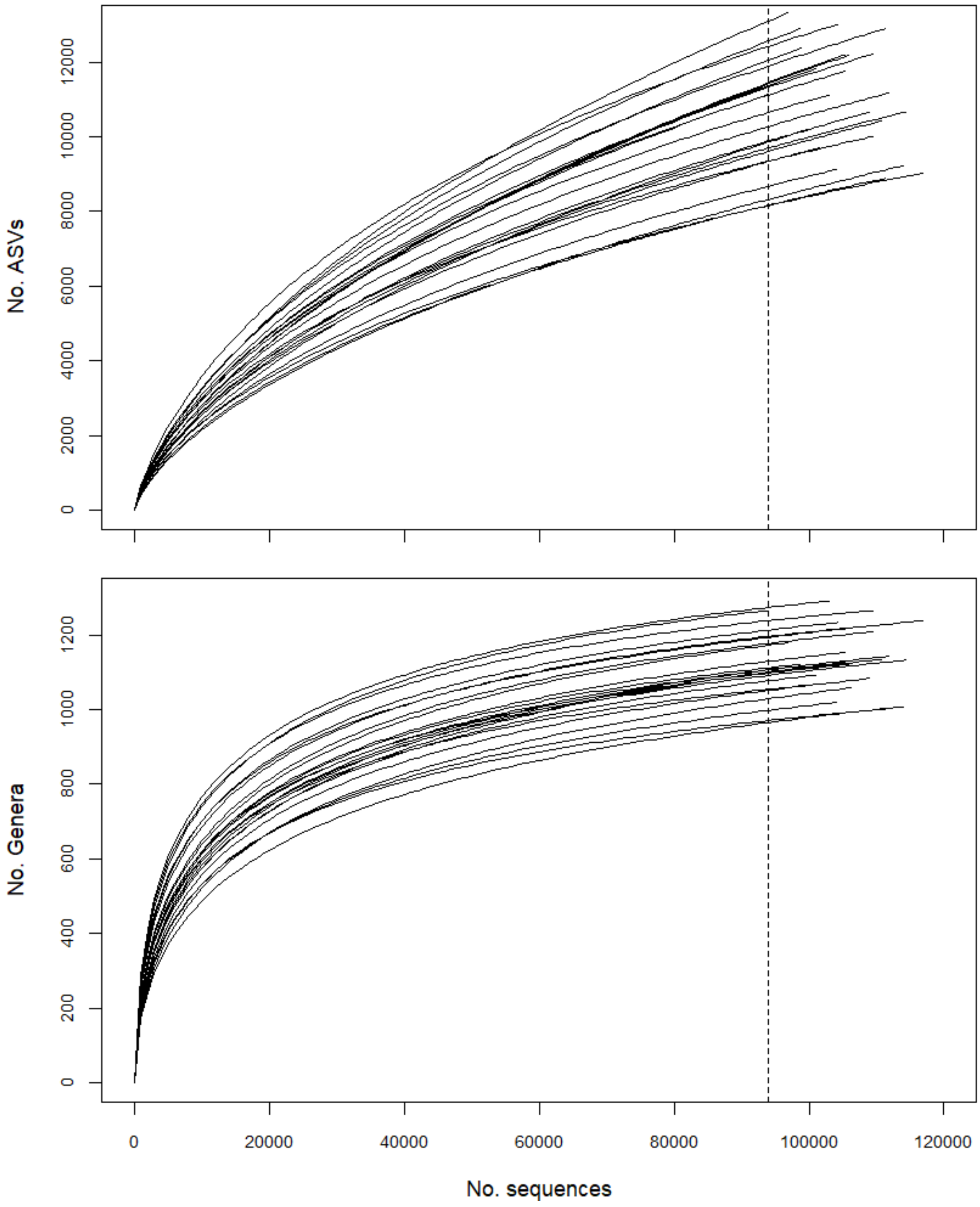

Supplementary figure 2. Rarefication curve for each sediment samples (n=21) showing the number of ASVs (top) and Genera (bottom) identified with increasing sequencing depth. Data set includes singletons, with a minimum of 93982 sequences obtained (dotted line).

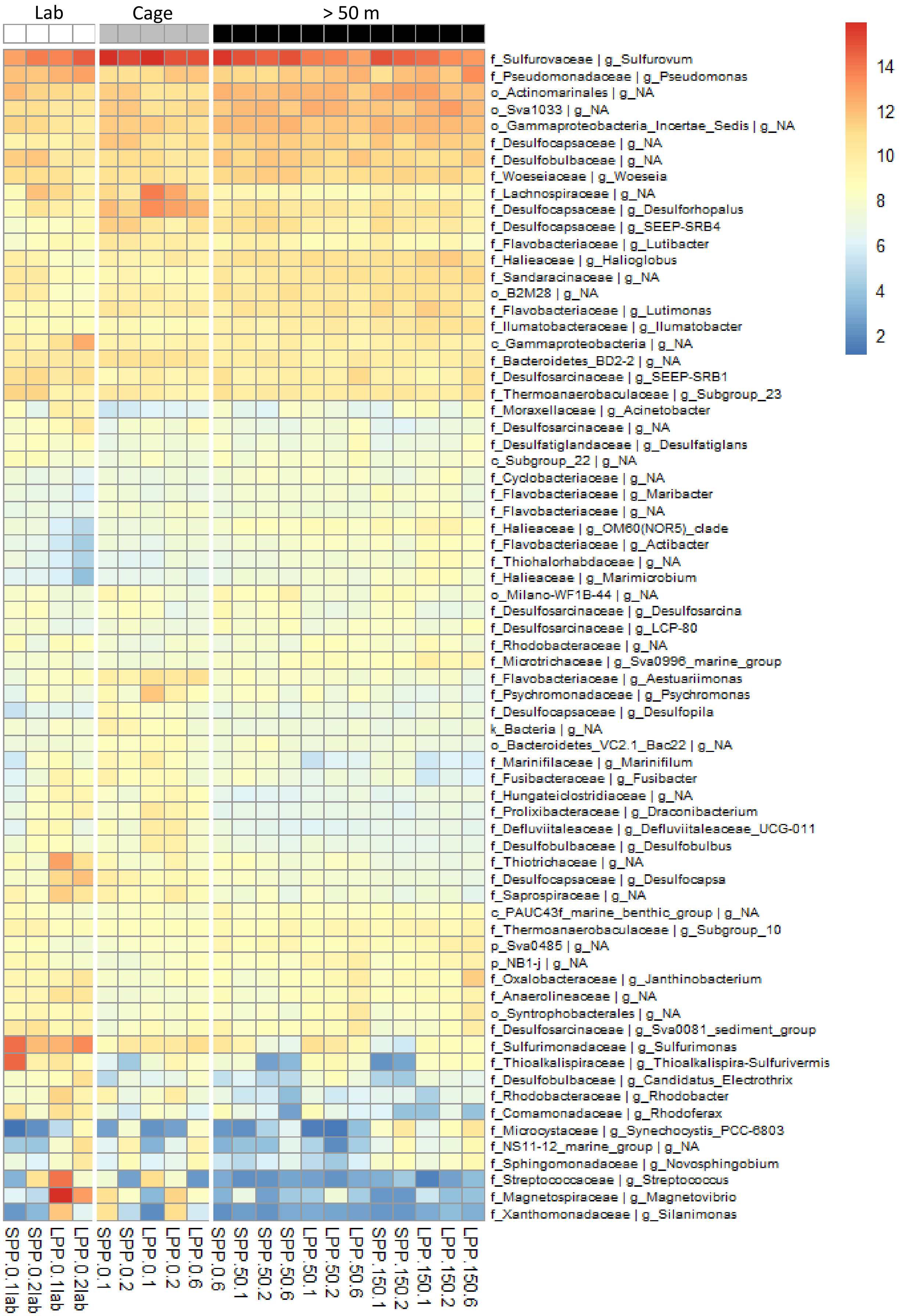

Supplementary figure 3. Top 70 most abundant genera for all fish farm sediments: Lab incubations (white), Cage (grey) and >50 m (black). Genus and next known taxonomic level is stated for each taxa (k=Kingdom, p=Phylum, o=Order, c=Class, f=family, g=Genus). Abundances were normalized with a variance stabilization transformation (blue to red scale bar). Sample code represent Production period: LPP=long, SPP=short, distance from cage: 0= cage, 50= 50m , 150= 150 m; sediment layers: 1= 0-0.5 cm, 2= 0.5-1.0 cm, 6= 3.0-5.0 cm. Sediment samples from laboratory incubations are additionally labelled as *lab*.

Supplementary Material

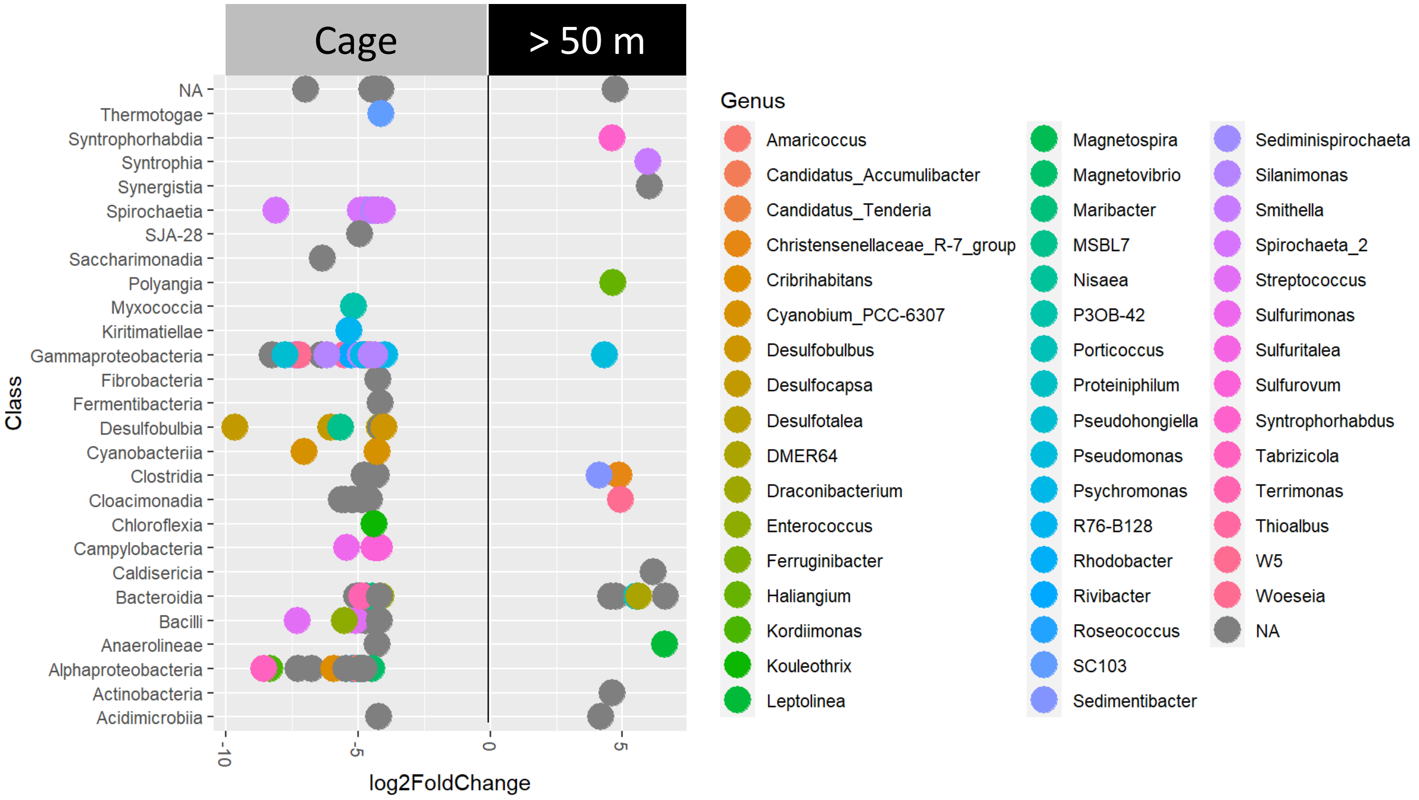

Supplementary figure 4. Differentially abundant 57 taxa (identified to Genus and Class level) determined with DESeq between the two PCoA sample clusters: cage vs >50 m away from cage. All ASVs present a LFC≥|4| and p<0.01.

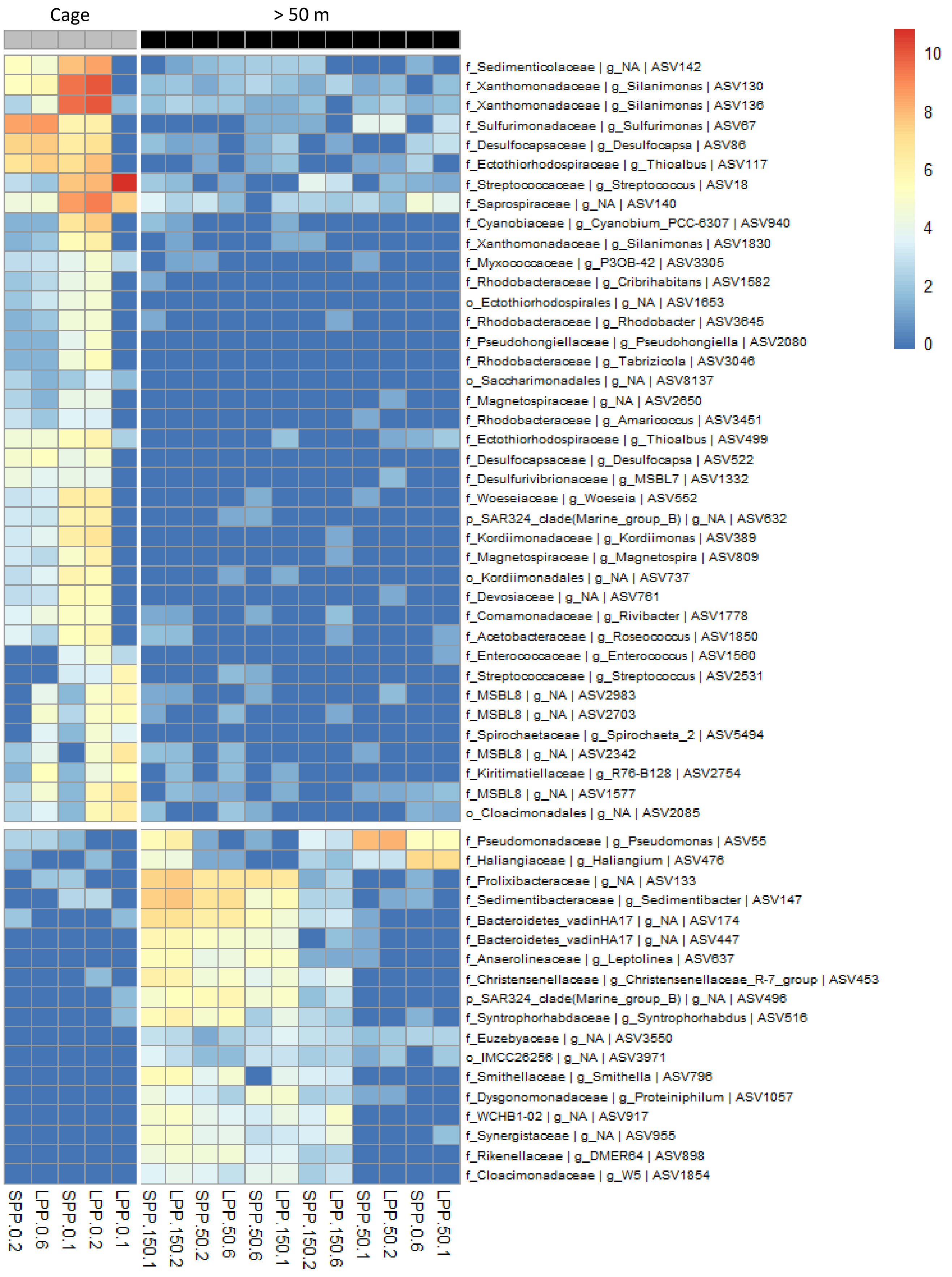

Supplementary figure 5. Abundance of 57 differentially abundant ASVs identified with DESeq (p<0.01) between PCoA groups. For Cage 37 ASVs with LFC>5 are listed while for >50 m 18 ASVs with LFC>4 are included. ASV numbers and lowest taxonomic classification are listed, full classification is present in supplementary table 3 (k=Kingdom, p=Phylum, o=Order, c=Class, f=family, g=Genus). Abundances were normalized with variance stabilizing transformation (blue to red scale bar). Sample code represent Production period: LPP=long, SPP=short, distance from cage: 0= cage, 50= 50 m , 150= 150 m; sediment layer: 1= 0-0.5 cm, 2= 0.5-1.0 cm, 6= 3.0-5.0 cm.

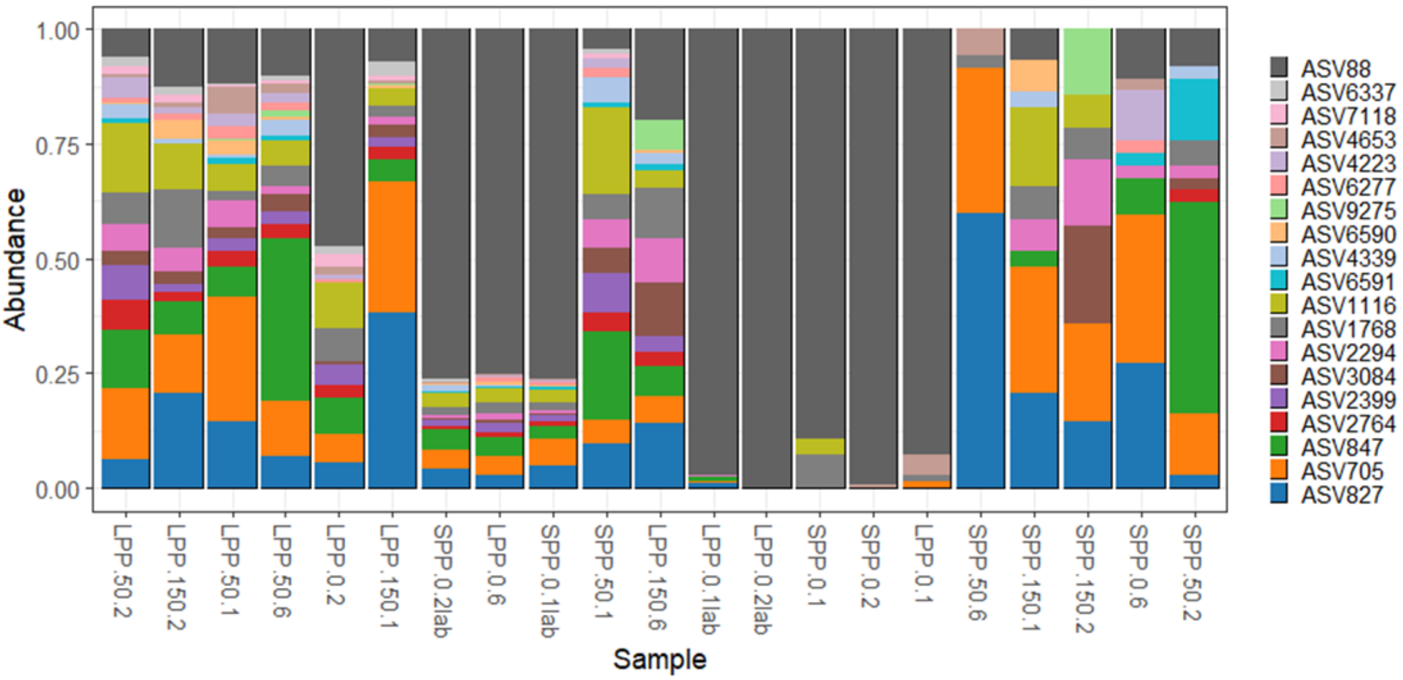

Supplementary figure 6. Relative abundance of the most abundant ASVs assigned to *Ca*. Electrothrix found in all sediment samples. Sample code represent Production period: LPP=long, SPP=short, distance from cage: 0= cage, 50= 50m , 150= 150 m; sediment layers: .1= 0-0.5 cm, .2= 0.5-1.0 cm, .6= 3.0-5.0 cm. Sediment samples from laboratory incubations are additionally labelled as *lab*. Samples are listed in the same order as in Figure 4 (main text).
